# AntiVIRmiR: A repository of host antiviral miRNAs and their expression along with experimentally validated viral miRNAs and their targets

**DOI:** 10.3389/fgene.2022.971852

**Published:** 2022-09-08

**Authors:** Anamika Thakur, Manoj Kumar

**Affiliations:** ^1^ Virology Unit and Bioinformatics Centre, Institute of Microbial Technology, Council of Scientific and Industrial Research (CSIR), Sector 39-A, Chandigarh, India; ^2^ Academy of Scientific and Innovative Research (AcSIR), Ghaziabad, India

**Keywords:** microRNA, target, expression, antiviral, viruses, human host, network, web server

## Abstract

miRNAs play an essential role in promoting viral infections as well as modulating the antiviral defense. Several miRNA repositories have been developed for different species, e.g., human, mouse, and plant. However, ‘*VIRmiRNA’* is the only existing resource for experimentally validated viral miRNAs and their targets. We have developed a *‘AntiVIRmiR’* resource encompassing data on host/virus miRNA expression during viral infection. This resource with 22,741 entries is divided into four sub-databases viz., ‘*DEmiRVIR’*, ‘*AntiVmiR’,* ‘*VIRmiRNA2’* and ‘*VIRmiRTar2’*. ‘*DEmiRVIR*’ has 10,033 differentially expressed host-viral miRNAs for 21 viruses. ‘*AntiVmiR*’ incorporates 1,642 entries for host miRNAs showing antiviral activity for 34 viruses. Additionally, ‘*VIRmiRNA2*’ includes 3,340 entries for experimentally validated viral miRNAs from 50 viruses along with 650 viral isomeric sequences for 14 viruses. Further, ‘*VIRmiRTar2*’ has 7,726 experimentally validated targets for viral miRNAs against 21 viruses. Furthermore, we have also performed network analysis for three sub-databases. Interactions between up/down-regulated human miRNAs and viruses are displayed for *‘AntiVmiR’* as well as *‘DEmiRVIR’*. Moreover, *‘VIRmiRTar2’* interactions are shown among different viruses, miRNAs, and their targets. We have provided browse, search, external hyperlinks, data statistics, and useful analysis tools. The database available at https://bioinfo.imtech.res.in/manojk/antivirmir would be beneficial for understanding the host-virus interactions as well as viral pathogenesis.

## Introduction

MiRNAs are short non-coding endogenous RNA (ncRNA) molecules with 18–23 nucleotides (nt) in length. They help in the post-transcriptional regulation of gene expression (Treiber et al., 2019). The miRNA biogenesis involves two types of processing pathways, viz. Canonical and non-canonical pathways (O’Brien et al., 2018). The canonical miRNA biogenesis includes four main processing steps. Firstly, it begins with the transcription of primary miRNA (pri-miRNA) from the genomic DNA (intronic, intergenic, or polycistronic loci) by RNA polymerase II in the nucleus. Secondly, the pri-miRNA consists of a hairpin structure that is further cleaved into ∼70 nt precursor miRNA (pre-miRNA) using the RNase III enzyme Drosha (Ha and Kim, 2014). This drosha enzyme is found in a multiprotein complex known as microprocessor that also include ds-RNA binding protein, DGCR8 (DiGeorge syndrome chromosomal region 8), also known as Pasha in *Caenorhabditis elegans* and Drosophila (Denli et al., 2004; Yeom et al., 2006). DGCR8 helps in the binding of dsRNA substrate by recognizing the “UGU” motif and helps drosha in the cleavage of pri-miRNA by binding to the basal “UG” motif (Han et al., 2004; Auyeung et al., 2013). Thirdly, this pre-miRNA is then exported to cytoplasm using Exportin-5 (XPO5)/RanGTP complex, where the hairpin loop is cleaved by the Dicer-transactivating response RNA-binding protein (TRBP) complex enzyme and results in miRNA duplex. Finally, the miRNA duplex is loaded onto the Argonaute protein (AGO)-containing RNA-induced silencing complex (RISC) complex to form a mature miRNA strand and its complementary passenger strand. The mature miRNA binds to the complementary strand of target mRNA and results in translational repression or mRNA degradation (Rupaimoole and Slack, 2017). However, in most cases, the passenger strand is degraded in the cytoplasm. For example, endonuclease C3PO degraded the passenger strand in C. elegans (Ye et al., 2011). But in some cases, the passenger strand plays a pivotal role in the cells where the mature strand works synergistically with the passenger strand (Shan et al., 2013; Yang et al., 2013).

However, non-canonical pathways are drosha/DGCR8 or dicer-independent pathways e.g., mirtrons produced from the pre-miRNA/introns during splicing (Ruby et al., 2007); 7-methylguanosine (m7G)-capped pre-miRNA exported to cytoplasm via PHAX-exportin one pathway (Xie M. et al., 2013). The first miRNA, lin-4 was discovered in 1993 by the Ambros and Ruvkun groups in *Caenorhabditis elegans* (Lee et al., 1993). Then, in 2000, a second miRNA, i.e. let-7 was also discovered in *Caenorhabditis elegans* (Reinhart et al., 2000). Since then, miRNAs have been discovered for organisms like human, worms, flies, mouse, rats, etc. (Lee and Ambros, 2001; Takada et al., 2006).

MiRNAs play an essential role in biological processes like cell division, stress conditions, cancer, proliferation, apoptotic cell death, viral infection, tumorigenesis, etc. (Huang et al., 2011). Host miRNA also help in replicating and propagating viruses by generating pri-miRNAs using RNA polymerase II (Grundhoff and Sullivan, 2011; Zhuo et al., 2013). miRNAs are also encoded from the viral genome and may assist in causing viral infection (Skalsky and Cullen, 2010; Bruscella et al., 2017). Host miRNAs can also target viral mRNAs that result in inhibiting the viral life cycle. For example, hsa-miR-296–5p targets IFN beta (Scagnolari et al., 2010) and VP1 and VP3 (Zheng et al., 2013), during Hepatitis C virus (HCV) and Enterovirus 71 (EV71) infections respectively. Contrary, a few host miRNAs e.g. hsa-miR-122, hsa-miR-151–5p, and hsa-miR-17–5p also result in promoting HCV infection (Li et al., 2017).

The host miRNAs play an essential role in the viral life cycle. Likewise, many viruses have their own miRNAs. The first viral miRNA was identified for Epstein-Barr virus (EBV) in 2004 (Pfeffer et al., 2004). Since then, miRNAs were identified in different viruses like Kaposi’s sarcoma-associated herpesvirus (KSHV) (Lin et al., 2012), Human cytomegalovirus (HCMV) (Pfeffer et al., 2005), Human immunodeficiency virus type 1 (HIV-1) (Su et al., 2018) etc. There are 1,308 viral miRNAs for 44 viruses reported in the ‘*VIRmiRNA’* database (Qureshi et al., 2014). Since then, numerous miRNAs and their isomiRs have been discovered for different species (Lagos-Quintana et al., 2002; Glazov et al., 2010). IsomiRs are miRNA sequences with variations in length at the 5’ or 3’ termini that result in nucleotide addition or deletion (Glogovitis et al., 2020).

Various computational resources have been developed that collect data for experimentally validated miRNA. The latest release of miRBase22 database has 38,589 miRNAs for 271 organisms. Likewise, there are many other databases for miRNA collecting data for different organisms. For example, *miRBase* (Kozomara et al., 2019), *miRviewer* (Kiezun et al., 2012), *miRNEST* (Szcześniak et al., 2012), *PMRD* (Zhang et al., 2010); *mirPub* (Vergoulis et al., 2015), *HMDD* (Li et al., 2014; Huang et al., 2019), *EpimiRBase* (Mooney et al., 2016), *HumiR* (Solomon et al., 2020), *miR2Disease* (Jiang et al., 2009), *miRCancer* (Xie B. et al., 2013) etc. Also, repositories have been developed for experimentally validated or predicted miRNA targets. For example, *targetHub* (Manyam et al., 2013)*, miRTarBase* (Hsu et al., 2011; Chou et al., 2018; Huang et al., 2020), *dbMTS* (Li et al., 2020), *maTE* (Yousef et al., 2019), *miRPathDB* (Backes et al., 2017; Kehl et al., 2020) etc. Similarly, there are web servers for differentially expressed miRNA developed using different miRNA profiling methods, namely, *miRExpress* (Wang et al., 2009), *miRGator* (Nam et al., 2008; Cho et al., 2013), *miRmine* (Panwar et al., 2017) etc.

Likewise, few repositories have been developed for viruses, viz., *ViTa* (Hsu et al., 2007) predicts targets for host miRNA on viruses; *Vir-Mir db* (Li et al., 2008) predicts viral miRNA hairpin; *vHoT* (Kim et al., 2012) database shows interactions between viral microRNA and host genomes. Also, our lab has developed a dedicated virus-encoded resource that has information for experimental viral miRNA and their targets, i.e. ‘*VIRmiRNA’* (Qureshi et al., 2014). Since, there are several studies for host miRNAs that are involved in viral infection. But still, a dedicated web resource for host miRNAs involved in viral diseases is lacking. Therefore, we have developed the ‘*AntiVIRmiR*’ database that encompasses information on host encoded antiviral miRNA with their expression and experimentally validated viral miRNA along with their targets. It includes four sub-databases, viz. DEmiRVIR, AntiVmiR, VIRmiRNA2 and VIRmiRTar2. DEmiRVIR sub-database incorporates differentially expressed host and viral miRNA data, AntiVmiR sub-database has host encoded antiviral and proviral miRNA data, VIRmiRNA2 sub-database provides experimentally validated viral miRNA data and VIRmiRTar2 sub-database includes experimentally validated viral miRNA target information. This resource would be helpful for the researchers that are focusing on host miRNA-virus interaction.

## Materials and methods

### Data collection

The literature search was carried out using the advanced search option of PubMed using the following query:

(((virus)OR(virus))AND((microrna)OR(mirna)))

Using this search, ∼2,300 articles were obtained as of March 2021. We focused majorly on screening miRNAs pertaining to important human viruses. After screening, ∼1,200 articles were retrieved to obtain the relevant information. Besides these, we also removed those articles that provide information about the predicted miRNA and some other research aspects of miRNAs. Finally, 620 articles were used to extract viral and antiviral miRNA information (Supplementary Figure S1). Further, we will update the data yearly or whenever sufficient data is available.

### Database organization

*‘AntiVIRmiR’* resource is divided into four sub-databases, namely, ‘*DEmiRVIR’,* ‘*AntiVmiR’* ‘*VIRmiRNA2’*, and ‘*VIRmiRTar2’.*

‘*DEmiRVIR’*' comprises data for differentially expressed miRNA found in the viral infection. It includes the following data: 1) DEmiRVIR ID, 2) virus, 3) taxonomy, 4) nomenclature, 5) miRNA name, 6) miRNA sequence, 7) miRNA expression (up/down), 8) organism, 9) cell line, 10) experimental method, 11) score, and (xiii) references.

‘*AntiVmiR’* module incorporates information regarding the host miRNA that acts mainly as an antiviral or sometimes as a proviral in different disease conditions. Following fields are incorporated in the sub-database, viz. 1) AntiVmiR ID, 2) virus, 3) taxonomy, 4) nomenclature, 5) miRNA name, 6) target gene, 7) uniprot id, 8) target organism, 9) target process, 10) cell line, 11) experimental method, 12) target region and 13) references.

‘*VIRmiRNA2’* sub-database provides data for experimentally validated viral miRNAs that are reported in literature. It incorporates information about the following fields: 1) VIRmiRNA2 ID, 2) virus, 3) taxonomy, 4) nomenclature, 5) viral miRNA name 6) viral miRNA sequence, 7) length, 8) GC content, 9) pre-miRNA, 10) arm, 11) cell line, 12) experimental method and 13) references.

‘*VIRmiRTar2’* sub-database has information about experimentally validated miRNA targets. It includes the following fields: 1) VIRmiRTar2 ID, 2) virus, 3) taxonomy, 4) nomenclature, 5) viral miRNA name, 6) target gene, 7) uniprot id, 8) target organism, 9) cell line, 10) experimental method, 11) target region, 12) target reference and 13) references.

### Network-based analysis

We have constructed the networks for three sub-databases, viz. ‘*DEmiRVIR’*, ‘*AntiVmiR’,* and ‘*VIRmiRTar2’.* Interactions are shown among viruses, miRNAs, and targets as input in the cytoscape software (Shannon et al., 2003). Different shapes and colors were used to represent the virus, miRNA and targets in different sub-databases. Using ‘*DEmiRVIR’* data*,* networks developed between human miRNA and virus. miRNA name is given in ellipse shape (pink color), upregulated human miRNA is denoted in diamond shape (red color); downregulated human miRNA in hexagon shape (purple color). While the human miRNAs found upregulated or downregulated in different studies are assigned octagon shape (green color). In *‘AntiVmiR’,* networks developed for human miRNA with different viruses where virus name is displayed in ellipse shape (red color) and human miRNA in octagon shape (pink color). While in ‘*VIRmiRTar2’,* networks developed between virus, viral miRNA and their targets. The virus name is provided in ellipse shape (red color), viral miRNA in octagon shape (pink color) and targets in triangular shape (green color).

### Implementation

**FIGURE 1:**
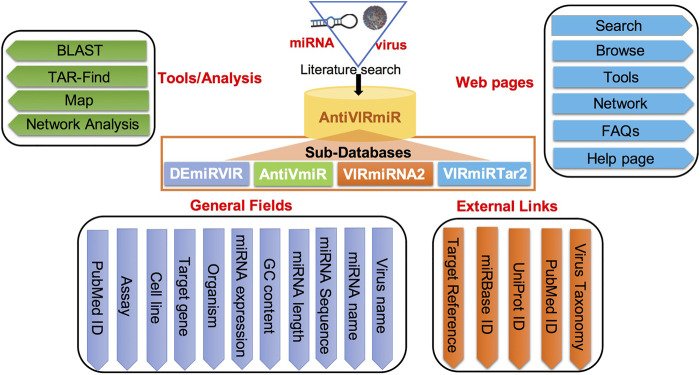
AntiVIRmiR architecture.

*‘AntiVIRmiR’* resource is developed user-friendly to access the data using different web pages like search, browse, analysis tools and network analysis. Further, a user manual is also provided on the help page to assist in data exploration. The database is designed using LAMP software that uses linux as an operating system and apache as a web server. The front-end of the web interface is developed using scripting languages viz.*,* Javascript, HTML, CSS and PHP. While back-end of the interface is implemented using MySQL database. The detailed architecture of *‘AntiVIRmiR’* is shown in Figure 1.

## Results

*‘AntiVIRmiR’* resource has been subdivided into four databases, viz. *‘DEmiRVIR’, ‘AntiVmiR’, ‘VIRmiRNA2’*, and *‘VIRmiRTar2’*.

### Database statistics

**FIGURE 2:**
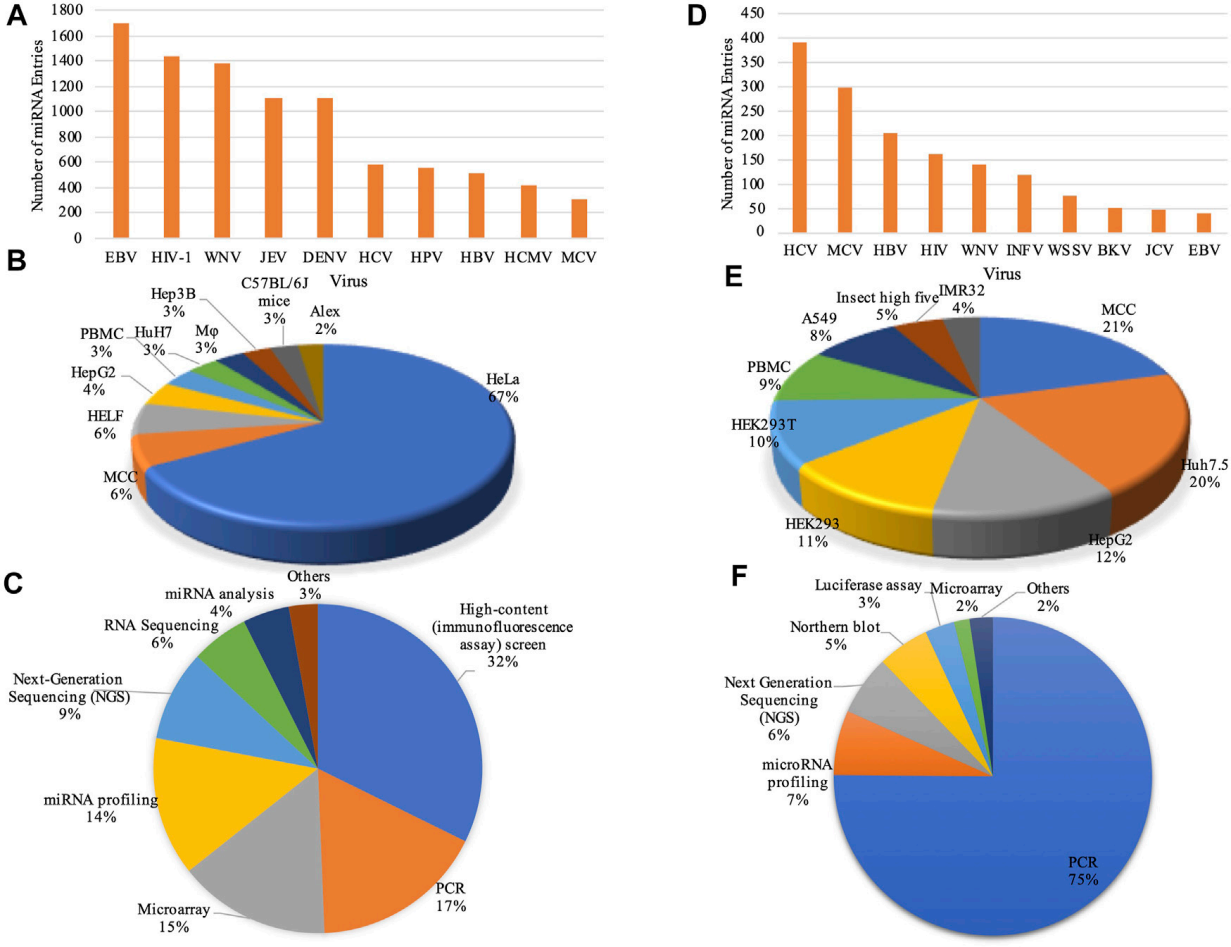
Data statistics: Bar graphs represent the number of entries in top 10 viruses for **(A)** *‘DEmiRVIR’* and **(D)** *‘AntiVmiR’*. Pie charts representing statistical distribution of cell lines in **(B)** *‘DEmiRVIR’* and **(E)** *‘AntiVmiR’* and experimental methods in **(C)** *‘DEmiRVIR’* and **(F)** *‘AntiVmiR’* (HCV, Hepatitis C virus; MCV, Merkel cell polyomavirus; HBV, Hepatitis B virus; HIV, Human immunodeficiency virus; WNV, West Nile Virus; INFV, Influenza A virus; WSSV, White spot syndrome virus; BKV, BK polyomavirus; JCV, JC polyomavirus; EBV, Epstein Barr virus; JEV, Japanese encephalitis virus; DENV, Dengue virus; HPV, Human papillomavirus and HCMV, Human cytomegalovirus).

We have provided exhaustive database statistics for each sub-category as depicted in Figure 2.

1. DEmiRVIR: *‘DEmiRVIR’* resource deals with the differentially expressed miRNA (DEmiRs) of hosts (primarily human) found against viruses. Currently, our database incorporates 10,033 miRNAs that are differentially expressed against 21 viruses. The maximum number of entries has been reported for EBV having 1705 entries followed by HIV-1 with 1,435 entries, West Nile Virus (WNV) with 1,386 entries, Japanese encephalitis virus (JEV) with 1,108 entries, Dengue virus (DENV) having 1,107 entries and so on (Figure 2A). The majority of these DEmiRs have been detected in cell lines like HeLa, MCC, HELF, HepG2, PBMC etc., as shown in Figure 2B. While, these DEmiRs are obtained by performing different experiments. The most commonly used experiments are High-content (immunofluorescence assay) screen, microarray, PCR, miRNA profiling, next-generation sequencing (NGS) etc. (Figure 2C).

2. AntiVmiR: This sub-database encompasses 1,642 entries reported for 34 viruses. The HCV has a maximum of 394 entries reported from 81 human miRNAs in different studies, followed by Merkel cell polyomavirus (MCV) with 297 entries encountered from 190 human miRNAs; Hepatitis B virus (HBV) having 204 entries from 42 human miRNAs and so on (Figure 2D). Different cell lines viz. MCC, Huh7.5, HepG2, HEK293 etc. Were used to test these miRNAs. Further, these human miRNAs were validated using experimental techniques like PCR, miRNA profiling, NGS, northern blot etc. (Figures 2E,F).

3. VIRmiRNA2: *‘VIRmiRNA2’* sub-database encompasses 3,340 viral miRNA entries from 50 viruses infecting different organisms. The majority of entries belongs to EBV with 604 viral miRNAs followed by 350 entries from Pseudorabies virus (PRV), 348 viral miRNAs from KSHV and other important viruses like HCMV, Rhesus lymphocryptovirus (RLCV), Herpes simplex virus 1 (HSV1) etc. As shown in Supplementary Figure S2A. These viral miRNA entries have been tested on different cell lines, like PK15, MDBK, IB-RS-2, HEK293T, MSB1, and Vero (Supplementary Figure S2B). Further, we found that experimental methods like PCR, deep sequencing, northern blot, next-generation sequencing (NGS) are the most commonly used methods for miRNA experiments, as depicted in Supplementary Figure S2C.

Isomirs: From 3,340 entries for the *‘VIRmiRNA2’* sub-database, we have found 650 unique viral miRNA isomeric sequences for 14 viruses. We have displayed a single viral miRNA entry with their isomeric sequence for individual viruses in Table 1. The maximum number of viral isomeric sequences have been reported for PRV with 276 isomirs for 26 viral miRNAs, followed by Rhesus rhadinovirus (RRV) and RLCV having 66 and 46 isomirs for 25 viral miRNAs respectively. Whereas, Herpes B virus has 11 viral miRNAs that include 57 isomirs. A complete list of viral miRNA isomeric sequences is given in Supplementary Table S1. In the case of individual viral miRNAs, the top six miRNAs belong to PRV, where prv-miR-11–1 has a maximum number of 40 entries, followed by prv-miR-9-5p, prv-miR-8-5p, prv-miR-5-5p, prv-miR-4-5p and prv-miR-7-5p with 32, 28, 25, 22 and 21 entries, respectively. Followed by Herpes B virus, hbv-miR-b8-5p and hbv-miR-b7-5p with 18 and 10 isomirs, respectively. Whereas, top hits for other viruses have less than 10 isomirs. For example, rlcv-miR-rl1-35–3p has seven isomirs; bfv-miR-bf2-3p, ebv-mir-bart16–3p and rrv-miR-rr1-7-5p having five isomirs; kshv-miR-K12-11–3p and rcmv-miR-r1-1 having four isomirs. Similarly, many miRNAs contain only single isomirs e.g., hcmv-miR-UL112–3p, hsv1-miR-H5-3p, mdv1-m11–5p, mdv2-m14–5p etc. These isomeric sequences are appended by # symbol after the miRNA sequence in the database.

**TABLE 1:** Representative isomeric miRNA sequences for 14 viruses.

S. No.	miRNA	miRNA sequence	Length	PMID
1	bfv-miR-bf1-3p	ucc​cug​aag​cca​uau​ccg​agg​c	22	24522910
		ucc​cug​aag​cca​uau​ccg​agg​cu	23	24522910
		ucc​cug​aag​cca​uau​ccg​agg​ca	23	24522910
		ucc​cug​aag​cca​uau​ccg​agg​u	22	24522910
		ucc​cug​aag​cca​uau​ccg​agg	21	24522910
2	blv-miR-b2-3p	ugc​gug​ucg​cuc​agu​cau​uuu	21	22308400
		ugc​gug​uca​cuc​agu​cau​uuu	21	22308400
3	dev-miR-d11–3p	gca​aaa​ggg​cag​ccu​ggg​cuc​uau	24	22492913
		aaaagggcagccugggcu	18	29704894
4	ebv-miR-bart7-3p	cau​cau​agu​cca​gug​ucc​agg​g	22	16557291,17604727, 16540699
		auc​aua​guc​cag​ugu​cca​gg	20	29425228
5	hbv-miR-b14rc-3p	agg​agg​ggu​cug​gga​gag​aag​gg	23	21543500
		agg​agg​ggu​cug​gga​gag​aag​g	22	21543500
		gga​ggg​guc​ugg​gag​aga​agg​g	22	21543500
		agg​agg​ggu​cug​gga​gag​aag	21	21543500
		gga​ggg​guc​ugg​gag​aga​a	19	21543500
6	hcmv-miR-UL148D	ucg​ucc​ucc​ccu​ucu​uca​ccg	21	31749099, 15782219
		ucg​ucc​ucc​ccu​ucu​uca​ccu	21	31749099
7	hsv1-miR-H2-3p	ccu​gag​cca​ggg​acg​agu​gcg​acu	24	21795359, 25535379
		cug​agc​cag​gga​cga​gug​cga	21	19656888
		cug​agc​cag​gga​cga​gug​cga​cu	23	19656888
		uga​gcc​agg​gac​gag​ugc​gac​u	22	19656888
8	kshv-miR-K12-11–3p	uua​aug​cuu​agc​cug​ugu​ccg​a	22	27611973, 30533200
		uua​aug​cuu​agc​cug​ugu​ccg	21	29425228
		uaa​ugc​uua​gcc​ugu​guc​cga	21	29425228
		augcuuagccuguguccg	18	29425228
		ccu​uaa​ugc​uua​gcc​ugu​guc​cg	23	29425228
9	mdv1-miR-m1/mdv1-m1-5p	ugc​uug​uuc​acu​gug​cgg​ca	20	16912324, 18842708
		ugc​uug​uuc​acu​gug​cgg​cau​u	22	24449754
		ugc​uug​uuc​acu​gug​cgg​cau​ua	23	24449754
10	mdv2-m14–5p	ugu​ggu​acg​gug​cac​ccu​gag​a	22	24449754
		gug​ugg​uac​ggu​gca​ccc​uga​ga	23	24449754
11	prv-miR-1-3p	ucu​cac​ccc​ugg​guc​cgu​cgc	21	22292087
		ucu​cac​ccc​ugg​guc​cgu​cgc​c	22	22292087
		cuc​uca​ccc​cug​ggu​ccg​ucg​c	22	22292087
		cuc​uca​ccc​cug​ggu​ccg​ucg	21	22292087
		ucu​cac​ccc​ugg​guc​cgu​cg	20	22292087
		ucu​cac​ccc​ugg​guc​cgu​c	19	22292087
12	rcmv-miR-orilyt-1	gac​ggg​guc​ucg​ggc​ucc​uga	21	20980502
		ccc​gga​gcu​cga​aac​ccg​guu​cg	24	20980502
		gac​ggg​guc​ucg​ggc​ucc​uga​c	22	20980502
13	rlcv-miR-rl1-1-3p	cuc​cgg​gcc​uga​aga​ggu​uga​c	22	16557291, 20219930
		cuc​cgg​gcc​uga​aga​ggu​uga	21	20219930
		cuc​cgg​gcc​uga​aga​ggu​ug	20	20219930
14	rrv-miR-rr1-1-3p	gcc​acc​gag​gau​gcg​guc​aau	21	20655562
		ggccaccgaggaugcggu	18	20655562, 17451774

4. VIRmiRTar2: *‘VIRmiRTar2’* section has 7,726 entries for experimentally validated miRNA targets obtained from 21 different viruses. The maximum number of targets have been reported for EBV, KSHV, Marek’s disease virus type 2 (MDV2), Marek’s disease virus type 1 (MDV1), HCMV, Bovine Leukemia Virus (BLV), Mouse gammaherpesvirus 68 (MGHV) with 3,846, 2,257, 859, 561, 107, 29, 25 entries respectively (Supplementary Figure S2D). While the remaining 42 entries have been reported for viruses like Herpes simplex virus (HSV), HIV-1, Simian virus 40 (SV40), JC polyomavirus (JCV) etc. In the database, ebv-miR-BART19–3p has a maximum of 372 entries, followed by ebv-miR-BART2-5p with 254 entries. In KSHV, kshv-miR-K12-11 has a maximum of 236 entries; kshv-miR-K12-4-3p with 223 entries; kshv-miR-K12-1 with 218 entries. In MDV, mdv2-miR-m16–5p and mdv1-miR-m6-5p have a maximum of 73 and 71 entries for respectively. Likewise, different cell lines have been used to identify miRNA targets e.g., BCBL-1, BC-1, BC-3, MSB1 and others (Supplementary Figure S2E), which includes cell lines like C666–1, B95-8, BJAB, Raji etc. Various experimental methods have been used for miRNA target identification. The most commonly used methods are Photoactivatable-Ribonucleoside-Enhanced Crosslinking and Immunoprecipitation (PAR-CLIP), Gene Set Enrichment Analysis (GSEA), PCR, luciferase assay, High Throughput Sequencing- Crosslinking and Immunoprecipitation (HITS-CLIP) (Supplementary Figure S2F). Whereas less commonly used methods for target identification are sequencing, RISC-Immunoprecipitation, microarray, ELISA etc.

### Network analysis

We have performed network analysis for three sub-databases to show the interactions among virus, miRNAs, and targets.

1. DEmiRVIR network analysis: In *‘DEmiRVIR’*, interaction analysis was performed for the human miRNA reported upon various viral infections. Human miRNAs express in host system either in human or cell line upon natural or artificial infection. In the database, different types of human miRNA belong to the same human miRNA family, e.g., let-7 (let-7a, let-7b, let-7c etc.), mir-130a, mir-18a, mir-146b etc. Similarly, different entries for 5p and 3p miRNA are shown, viz. mir-142–3p, mir-142–5p, mir-181a-2-3p etc. Therefore, we have used the original name of human miRNA by excluding its type, arm, e.g., let-7a and let-7b will be denoted as let-7, mir-142–3p will be used as mir-142. After this nomenclature, redundant entries were excluded from the analysis. Finally, 1,021 entries were left for upregulated miRNAs (507 unique) and 996 for downregulated miRNAs (426 unique). We have analyzed only those human miRNAs whose expression change during infection of five or more viruses in ‘DEmiRVIR’ sub-database. We found that 52 upregulated and 59 downregulated human miRNAs e.g., hsa-miR-146 was upregulated during 10 viral infections, followed by hsa-let-7, hsa-miR-18, hsa-miR-182 in eight viral infections and many more.

**FIGURE 3:**
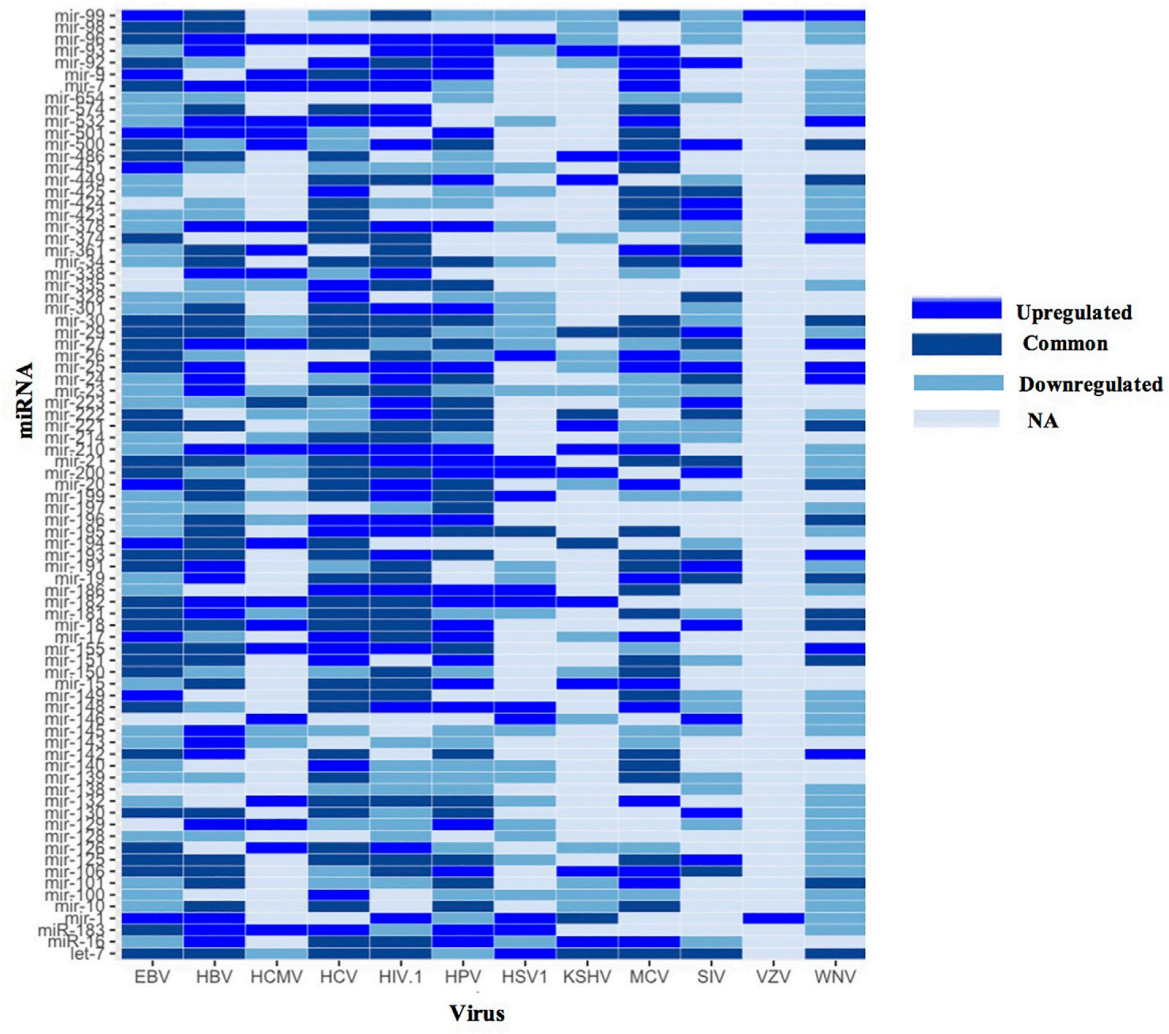
Heatmap showing human miRNA expression reported in different viruses (EBV, Epstein Barr virus; HBV, Hepatitis B virus; HCMV, Human cytomegalovirus; HIV, Human immunodeficiency virus; HPV, Human Papillomavirus; HSV1, Herpes simplex virus one; KSHV, Kaposi sarcoma-associated herpesvirus; MCV, Merkel cell polyomavirus; SIV, Simian immunodeficiency virus; VZV, Varicella-zoster virus and WNV, West Nile Virus).

From the 52 upregulated and 59 downregulated human miRNAs during viral infections (of five or more viruses), we found 30 human miRNAs were both upregulated and downregulated in different studies. While the remaining 22 upregulated human miRNAs were also found downregulated in less than five viral infections. Whereas, from the remaining 29 downregulated human miRNAs, namely, hsa-miR-128, hsa-miR-138 and hsa-miR-654 were not found in upregulated human miRNA data. In Supplementary Figure S3, the bar graph depicts the number of human miRNAs that are upregulated, downregulated or both in five or more viral infections. The maximum 50 differentially expressed human miRNAs have been reported for viral infections of EBV and HIV-1, followed by HBV (47), HCV (45), HCMV 32) etc. While, human miRNAs that were both upregulated and downregulated in different studies were found to have a maximum 21 in case of HCV followed by EBV (18), HBV 16) etc. Further, there is no human miRNA found that is both upregulated and downregulated during HCMV infection. Whereas, there are only two upregulated human miRNAs found during Varicella-zoster virus (VZV) infection. Using this data, we have developed a heatmap as depicted in Figure 3. Heatmap represents the human miRNA expression as upregulated, downregulated or both during different viral infections as reported in the literature. We found hsa-miR-25 and hsa-miR-210 human miRNAs were upregulated in maximum seven viral infections followed by hsa-miR-96 and hsa-miR-532 during six viral infections. Contrary, hsa-miR-145 human miRNA was downregulated in eight viral infections followed by hsa-miR-23 in 7, hsa-miR-654, hsa-miR-100 and hsa-miR-139 in six viral infections respectively. There are many human miRNAs e.g., let-7, hsa-miR-30, hsa-miR-29, hsa-miR-125 etc. That were both upregulated and downregulated for the same viral infection in different studies.

**FIGURE 4:**
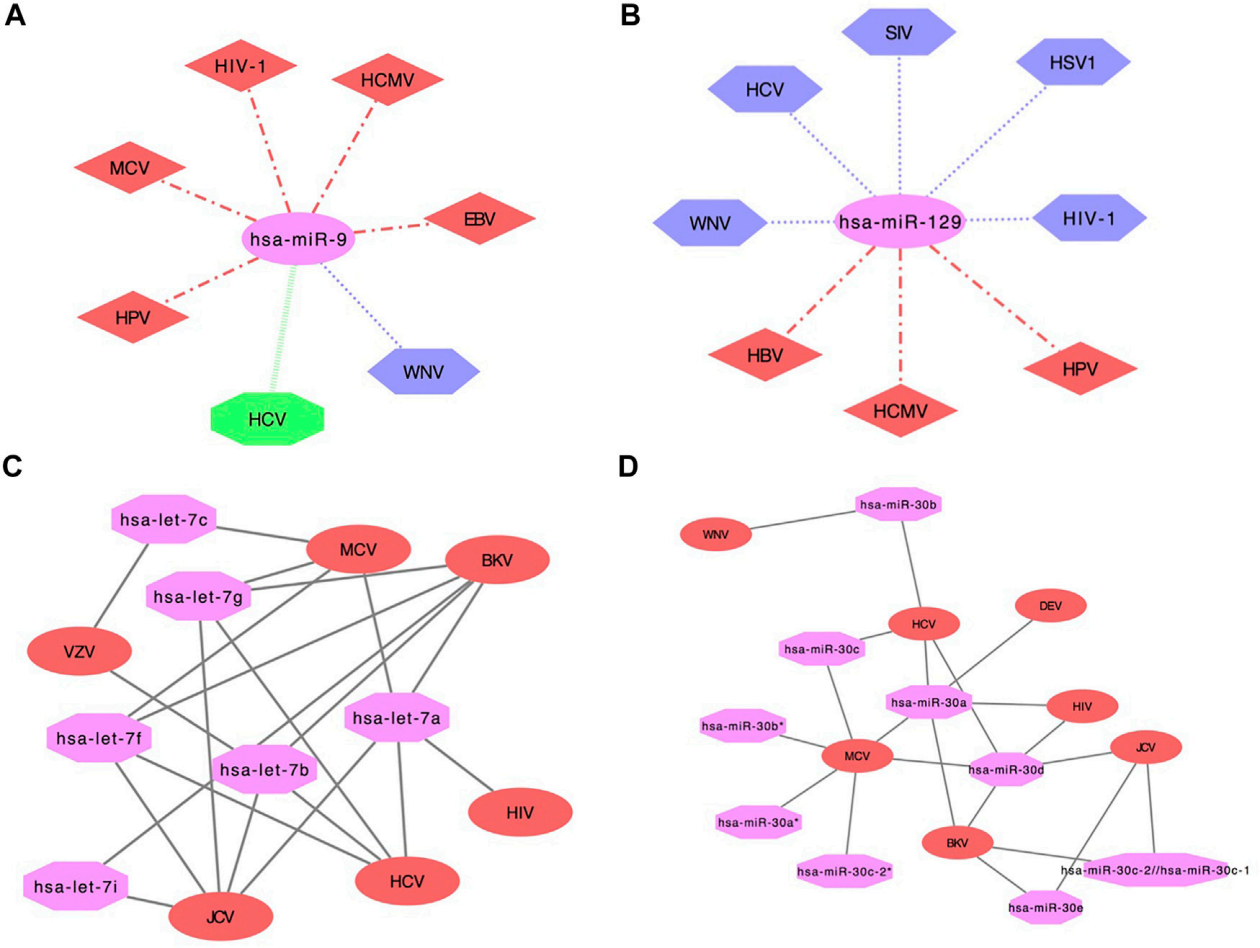
Network showing human miRNA expression reported in different viruses in *‘DEmiRVIR’* **(A)** hsa-miR-9 and **(B)** hsa-miR-129 (Viruses with hexagon shape (purple color) represent downregulation, diamond shape (red color) represents upregulation, octagon shape (green color) represents viruses found common in both upregulation as well as downregulation in different studies and miRNA is represented in ellipse shape (pink color)). Network interaction analysis for *‘AntiVmiR’* **(C)** let-7 and **(D)** mir-30. Virus name is written in ellipse shape (red color) and miRNA name in octagon shape (pink color).

Additionally, we have also developed networks using cytoscape software. In the network analysis, miRNA name is displayed in pink color (ellipse shape), whereas viral infections that help in the upregulation of human miRNA are denoted in red color (diamond shape) and downregulation in purple color (hexagon shape). Moreover, viral infections are assigned green color (octagon shape) if human miRNA expression was both upregulated and downregulated in different studies. For example, hsa-miR-9 human miRNA upregulated in five viral infections i.e., Human papillomavirus (HPV), MCV, HIV-1, HCMV, EBV and downregulated in WNV. Further, hsa-miR-9 human miRNA was both upregulated and downregulated in HCV in different studies (Figure 4A). Likewise, hsa-miR-129 human miRNA was found upregulated in three viral infections i.e., HBV, HCMV, HPV and downregulated in five viral infections i.e., WNV, HCV, Simian immunodeficiency virus (SIV), HSV1 and HIV-1. But in none of the viral infections, hsa-miR-129 human miRNA was found both upregulated and downregulated, as shown in Figure 4B. Further, hsa-let-7 human miRNA was upregulated in HSV1, downregulated in HPV and HCMV. Whereas it was upregulated or downregulated in eight viral infections viz., MCV, HBV, WNV, EBV, KSHV, SIV, HIV-1 and HCV (Supplementary Figure S4A). Moreover, hsa-miR-128 human miRNA was downregulated in five viral infections viz., EBV, HSV1, HBV, WNV and HIV-1 as displayed in Supplementary Figure S4B.

2. AntiVmiR Network analysis: *‘AntiVmiR’* interaction has been performed for human miRNAs that were found common in different viruses. We have developed 82 interaction networks for antiviral human miRNA for 28 viruses using the cytoscape software available on the web server. In the network analysis, the human miRNA name is written in octagon shape having magenta color. While the virus name is displayed in an ellipse shape having red color. The maximum number of viruses has been reported for hsa-miR-21 and hsa-miR-181 that show its antiviral effect against 9 viruses. hsa-miR-125 and hsa-miR-155 were reported against eight viruses, followed by hsa-miR-30 and hsa-miR-221 with seven viruses and so on. While building the network, we have shown common viruses for different variants of miRNA in one class. e.g., miRNA hsa-let-7a, 7b, 7c, 7f, 7g, 7i belongs to the let-7 family (Figure 4C). The let-7 family has been found in six viruses, where let-7a was found in BK polyomavirus (BKV), MCV, JCV, HCV and HIV; followed by let-7b (BKV, VZV, JCV and HCV), let-7f (MCV, BKV, HCV and JCV) and let-7g (JCV, HCV, BKV and MCV) in four viruses, let-7c (VZV and MCV) and let-7i (JCV and BKV) in two viruses. Likewise, the hsa-miR-30 family has variants like 30a, 30b, 30c*. hsa-miR-30a (HCV, MCV, Duck enteritis virus (DEV), BKV and HIV) and hsa-miR-30 days (BKV, HCV, MCV, HIV and JCV) were found in five viruses; hsa-miR-30b (WNV and HCV), hsa-miR-30c (MCV and HCV) and hsa-miR-30e (BKV and JCV) were found in two viruses. Whereas, hsa-miRmir-30a*, hsa-miR-30b* and hsa-miR-30c-2* were found in MCV (Figure 4D).

3. VIRmiRTar2 Network analysis: We have also performed network-based analysis on viral miRNA target data for 20 viruses using Cytoscape software. We have used viruses, viral miRNAs and their target as input in the software for building a network. Different shapes and colors were used to represent the virus (ellipse shape having red color), miRNA (octagon shape with pink color), and target (triangle shape in green color). The network analysis for HIV and BLV has been shown in Supplementary Figure S5. The viral miRNA for HIV further interacts with their respective target (Supplementary Figure S5A). There are three viral miRNAs for BLV that have some common targets like TLR9, C4A, COLEC12 in blv-miR-B1-5p and blv-miR-B4-5p (Supplementary Figure S5A). Therefore, users can view viral miRNA target interaction analysis for different viruses on the web server.

### Web server interface

We have provided a search and browse-by virus option to retrieve the data from each of the four different sub-databases.

**FIGURE 5:**
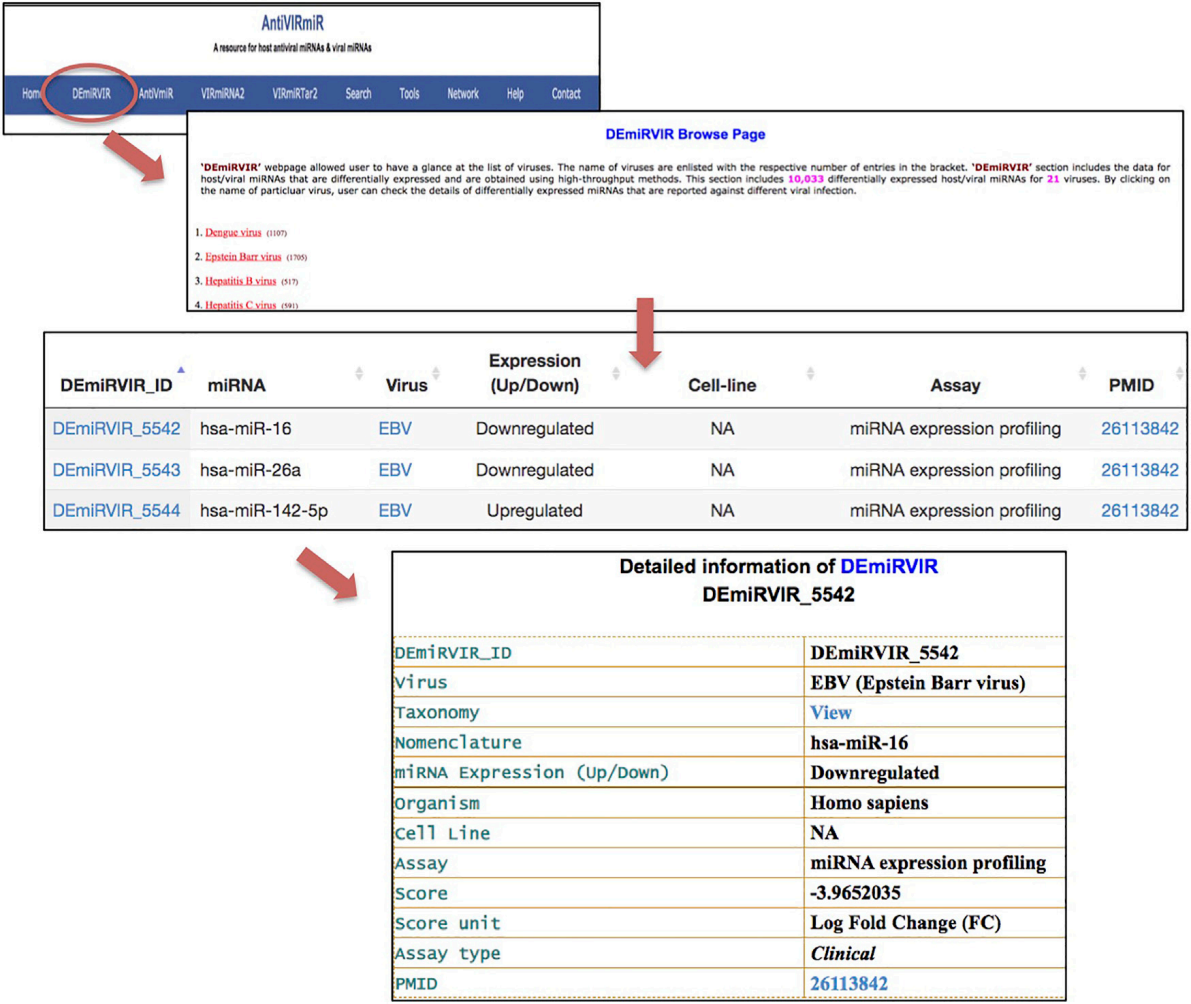
‘AntiVIRmiR’ resource overview that includes a browse page, individual search output and detailed information of a miRNA*.*

1. Database browsing: Users can browse each sub-database of *‘AntiVIRmiR’* by selecting any virus from the given list. The virus name is written with the number of miRNAs for that virus, e.g., EBV has 1705 entries in the *‘DEmiRVIR’* sub-database. Users can click on the virus name, and a list of all the miRNAs from that virus will be displayed. By clicking on miRNA ID, the user can view more details. Further, we have also provided an external link for NCBI Taxonomy browser, Uniprot, miRBase and PubMed (Figure 5).

2. Database search: Users can select any of the four sub-databases to search against the given keyword in the search option. The search output provides information about different aspects, i.e., miRNA, virus, miRNA expression, nomenclature, sequence, length, GC content, cell line, assay and pubmed. We have also provided sorting and filtering functionality in the search output. By clicking on the miRNA ID, users can view the complete details of that entry.

3. Analysis Tools: We also have integrated tools in the database like BLAST, Map and TarFind for miRNA sequence analysis. *AntiVIRmiR-BLAST* tool matches the user-provided miRNA sequence against the viral miRNA sequences (*‘VIRmiRNA2’*) that are already reported in the database. The output shows both tabular and text alignment of the matching miRNA sequences along with their alignment score. Map tool displays the list of perfectly matching miRNAs available in the database against the given input sequence. *Map* tool will help the user find how many miRNAs are available in the database against the user-provided sequence. *TarFind* tool identifies the target genes against the user-provided miRNA or seed sequence in the human reference gene sequence.

## Discussion

Viral miRNA and host miRNA play an essential role in viral pathogenesis during infection. It helps in promoting viral replication by cell survival, proliferation, or modulating the immune response (Skalsky and Cullen, 2010). Viral miRNAs have been discovered as post-transcriptional gene regulators for the host and viral genes. They can bind to single or multiple targets, also known as multiplicity, as reported in different studies (John et al., 2004; Qureshi et al., 2014). For example, ebv-miR-BART19–3p targets RBM47 and UHRF1BP1L (Gottwein et al., 2011); kshv-miR-K12-4-3p targets ZFYVE20, MACROD2, CASP3, MBNL3 and C9orf41 etc. (Gottwein et al., 2011). Simultaneously, there were reports of cooperativity in which multiple viral miRNAs target a gene (John et al., 2004). Like TPM1 (Tumor suppressor Protein tropomyosin 1) has been targeted by multiple KSHV viral miRNAs like kshv-miR-K12-1, kshv-miR-K12-2, kshv-miR-K12-3, kshv-miR-K12-4 (Kieffer-Kwon et al., 2015). In another example, THBS1 (Thrombospondin 1) is also targeted by multiple KSHV viral miRNAs resulting in the reduction of its activity at mRNA and protein level (Samols et al., 2007). However, there are genes that can be targeted by different viral miRNAs encoded by different viruses, e.g. MICB (major histocompatibility complex class I-related chain B) gene is targeted by ebv-miR-BART2-5p, hcmv-miR-UL112 and kshv-miR-K12-7 that result in the reduction of stress-induced natural killer (NK) cell mediated killing of infected cells (Nachmani et al., 2009). Similarly, human miRNAs viz. hsa-miR-122, hsa-miR-199a, hsa-miR-30, hsa-miR-573, hsa-miR-411 help in HCV replication are also found to influence the viral infection (Lanford et al., 2010; Kumar, 2011; Li et al., 2017). Likewise, hsa-miR-155 results in EBV-regulated signal transduction pathways by targeting genes like BACH1, ZIC3, HIVEP2, CEBPB etc. (Yin et al., 2008). Similarly, hsa-miR-199a-3p (Santhakumar et al., 2010) and hsa-miR-613 (Wang et al., 2017) were found downregulated in HCMV infection targeting AKT1 and ARG2 genes, respectively. Few repositories have been developed that contain viral miRNA information for different organisms, e.g. *‘miRBase’* (Kozomara et al., 2019)*, ‘ViTa’* (Hsu et al., 2007)*, ‘Vir-Mir db’* (Li et al., 2008) and *‘VIRmiRNA’* (Qureshi et al., 2014). In this study, we provide an integrated platform for human/viral miRNA with their expression and targets.

‘DEmiRVIR’ sub-database has been developed to provide the list of differentially expressed miRNAs (DEmiRs) of human and viruses. These miRNAs have been identified using various profiling methods like RT-PCR, NGS-based techniques etc. This sub-database incorporates unique 507 and 426 human miRNAs that are upregulated and downregulated respectively. While 258 human miRNAs were both upregulated and downregulated during viral infections as reported in different studies. We found that human miRNAs hsa-miR-25 and hsa-miR-210 were upregulated in maximum seven viral infections. These miRNAs are shown to play important role in cancer cell proliferation, metastasis (Wong et al., 2012; Peta et al., 2018), and also involved in KSHV (Viollet et al., 2017) and HIV infections (Modai et al., 2019). Likewise, hsa-miR-145 was downregulated during eight viral infections. This miRNA helps in oncogene expression at pre- and post-transcriptional level (Ye et al., 2019) and was downregulated in MCV + ve samples associated with non-small cell lung cancer (NSCLC) (Lasithiotaki et al., 2017). Likewise, hsa-miR-23 was downregulated in seven viral infections and differentially expressed in burkitt lymphomas (BL) vs. extramedullary plasmacytoma (EMPC) (Ambrosio et al., 2017). This miRNA can be used as a biomarker in molecular diagnosis (Fatmi et al., 2020). Further, hsa-let-7 was both upregulated and downregulated during eight viral infections reported in different studies. For example, hsa-let-7a upregulated in EBV-positive cells (Mansouri et al., 2014) and downregulated in SNT16 cells, which is an EBV-infected cell line (Alles et al., 2016). Likewise, hsa-let-7e upregulated in peripheral blood mononuclear cells (PBMCs) of chronic hepatitis C (CHC) patients compared with the healthy donors HCV (Chang et al., 2014) and downregulated in human hepatoma cells (Steuerwald et al., 2010).

Few repositories have been developed that encompass miRNA expression data viz. *‘mirEX 2.0’* resource for plant miRNA expression profiling (Zielezinski et al., 2015), *‘HMED’* database for human miRNA expression profiling that includes tissue and disease-specific miRNAs (Gong et al., 2014), *‘PmiRExAt’* is a plant miRNA expression database (Gurjar et al., 2016), *‘miRmine’* is a resource for human miRNA expression profiling (Panwar et al., 2017) etc. The *‘miR2Disease’* database only contains information on hepatitis B and C virus showing descriptions of the miRNA expression pattern (Jiang et al., 2009). Therefore, *‘DEmiRVIR’* is the first dedicated resource representing miRNA expression data for 21 viral infections.

The *‘AntiVmiR’* sub-database holds information about the human and other host miRNAs that shows the antiviral effect. hsa-miR-21 is the most explored miRNA found in 9 viruses viz.*,* BKV, HBV, HCV, HIV, HPV, JCV, KSHV, MCV and SIV. hsa-miR-21 plays an essential role in causing viral infections associated with cancer (Lasithiotaki et al., 2017). It can be used as a biomarker for HBV infection, a risk factor in causing hepatocellular carcinoma (HCC) (Liu et al., 2011). hsa-miR-155 is the second most explored miRNA that shows its antiviral activity against eight viruses viz.*,* EBV, HBV, HIV, HSV1, MCV, SFV, VSV and WNV. This miRNA plays an important role in inflammation, autoimmunity, cancer etc. And can be used as a disease biomarker (Alivernini et al., 2017). Further, hsa-miR-155 also helps control hypoxia-inducible factor 1α (HIF-1α) and promotes angiogenesis (Yang et al., 2016). Further, a few miRNAs were also reported in the literature that shows their proviral activity against HCV, viz. hsa-miR-122, hsa-miR-135a, hsa-miR-151–5p, hsa-miR-514, hsa-miR-548, hsa-miR-607, hsa-miR-191*, hsa-miR-17–5p, hsa-miR-589, and hsa-miR-657 (Kim et al., 2016; Li et al., 2017; Sodroski et al., 2019). Sodroski C et al. study suggests that host factors like RIPK2 (receptor-interacting serine/threonine kinase 2), MYD88 (myeloid differentiation primary response 88) result in the proviral effect of hsa-miR-135a on HCV (Sodroski et al., 2019). Kim GW et al., shows the proviral activity of hsa-miR-122 by inhibiting GLD-2, a non-canonical cytoplasmic poly(A) polymerase (Kim et al., 2016). Therefore, *‘AntiVmiR’* is a dedicated repository that provides the information on human and other host miRNA showing antiviral effects.

*‘VIRmiRNA2’* sub-database is an exhaustive resource for viral miRNA encompassing 3,340 viral miRNA entries for 50 viruses. In the previous repository, *‘VIRmiRNA’* contains 1,308 viral miRNA entries from 44 viruses, while *‘miRBase’* has 532 viral miRNA entries from 34 viruses (Qureshi et al., 2014). We have incorporated six new viruses in the database, i.e., Hepatitis A virus (HAV), Hepatitis B virus (HeBV), Japanese macaque rhadinovirus (JMRV), Murine (Mouse) cytomegalovirus (MCMV), Simian foamy virus (SFV) and Tiger frog virus (TFV). The current repository has about a 2.5-fold increase than *‘VIRmiRNA’* and a 6-fold increase than the miRBase data.

The *‘VIRmiRNA2’* sub-database also includes isomeric viral miRNA sequences for 14 viruses. Isomirs are miRNA variants generated from a single miRNA having variations in length and sequence. Majority of the isomirs were derived from the argonaute proteins, as reported in different studies (Martí et al., 2010; Cloonan et al., 2011). In our database, PRV has the maximum number of isomirs detected from the sequencing library using the infected PK-15 cells. These viral isomirs have a high sequence identity except terminal sequences/repeats (Wu et al., 2012). Likewise, viral isomirs for RRV were also identified using deep sequencing by analyzing the RNA expression using RRV-infected B-cell lymphoma and retroperitoneal fibromatosis tissues and their expression level was checked using 293T cells (Umbach et al., 2010). Therefore, *‘VIRmiRNA2’* provides exhaustive information about the viral miRNAs and isomirs.

*‘VIRmiRTar2’* sub-database holds updated information of experimentally validated targets of viral miRNAs from 21 viruses. Gottwein E et al. study reported majority of target genes, i.e. 5060 targets for KSHV and EBV (Gottwein et al., 2011). The study suggested that KSHV miRNAs target >2000 genes during KSHV pathogenesis, while EBV miRNAs also target >2,900 of these genes by using distinct binding sites. Further, Parnas O et al. reported a total of 1,413 targets including 554 for MDV1 and 859 for MDV2 (Parnas et al., 2014). This study performs mRNA analysis targeted by miRNAs expressed in the chicken T-cell line, i.e. MSB1, which naturally gets infected with MDV-1 and MDV-2. Moreover, we identified CASP3 (Caspase 3) targeted by a maximum 23 miRNAs encoded by three viruses, viz., EBV, KSHV and MDV2. Harold C et al. study showed that CASP3 is a putative target for one or more EBV miRNAs. They cloned the 3′-UTR region of CASP3 using a dual-luciferase reporter vector and further co-transfected HEK293T cells with EBV miRNAs (Harold et al., 2016). Likewise, THBS1 (thrombospondin 1) is targeted by 17 miRNAs of KSHV. Samols MA et al. study revealed that THBS1 is down-regulated more than10-fold by KSHV miRNAs that leads to the reduction of TGF-beta activity (Samols et al., 2007). Repositories have been developed for experimentally validated miRNA targets, e.g. ‘miRTarBase’, that contain target information for only three viruses, i.e. EBV, KSHV and HCMV (Huang et al., 2020). ‘TargetScan’ predicted the miRNA target sites in the mammalian mRNAs (Agarwal et al., 2015). Likewise, there are few resources for predicted targets of viral miRNAs like ‘ViTa’ (Hsu et al., 2007), ‘RepTar’ (Elefant et al., 2011), ‘vHOT’ (Kim et al., 2012), ‘Vir-Mir db’ (Li et al., 2008) etc. But these resources only furnish information about the predicted targets of viral miRNA. In this study, we are providing experimentally validated targets of viral miRNAs encoded by 21 viruses.

## Conclusion

*‘AntiVIRmiR’* is a miRNA repository that includes the human and virus miRNA expression data during the viral infection. In this resource, we have provided exhaustive information about the differentially expressed human and viral miRNAs and miRNAs having antiviral activity. We have also provided information about the experimentally validated viral miRNAs along with the isomirs and their targets. The resource would assist in understanding the host-virus interactions and may also be useful in developing miRNA-based therapeutics.

## Data Availability

The datasets presented in this study can be found in online repositories. The names of the repository/repositories and accession number(s) can be found in the article/Supplementary Material.
