# Supplementary material for: AntiVIRmiR: A repository of host antiviral miRNAs and their expression along with experimentally validated viral miRNAs and their targets
Source: Front Genet. 2022 Sep 8;13:971852. doi: 10.3389/fgene.2022.971852 (PMC9493126; doi:10.3389/fgene.2022.971852)
Supplement: Supplementary file 1 [file DataSheet1.pdf]

AntiVIRmiR: A repository of host antiviral miRNAs and their expression along with experimentally validated viral miRNAs and their targets

Anamika Thakur[1,2] and Manoj Kumar[1,2]*

[1]Virology Unit and Bioinformatics Centre, Institute of Microbial Technology, Council of Scientific and Industrial Research (CSIR), Sector 39-A, Chandigarh-160036, India

[2]Academy of Scientific and Innovative Research (AcSIR), Ghaziabad-201002, India

* To whom correspondence should be addressed. Tel: +91 172 6665158; Fax: +91 172 2690585; Email: manojk@imtech.res.in

Supplementary information

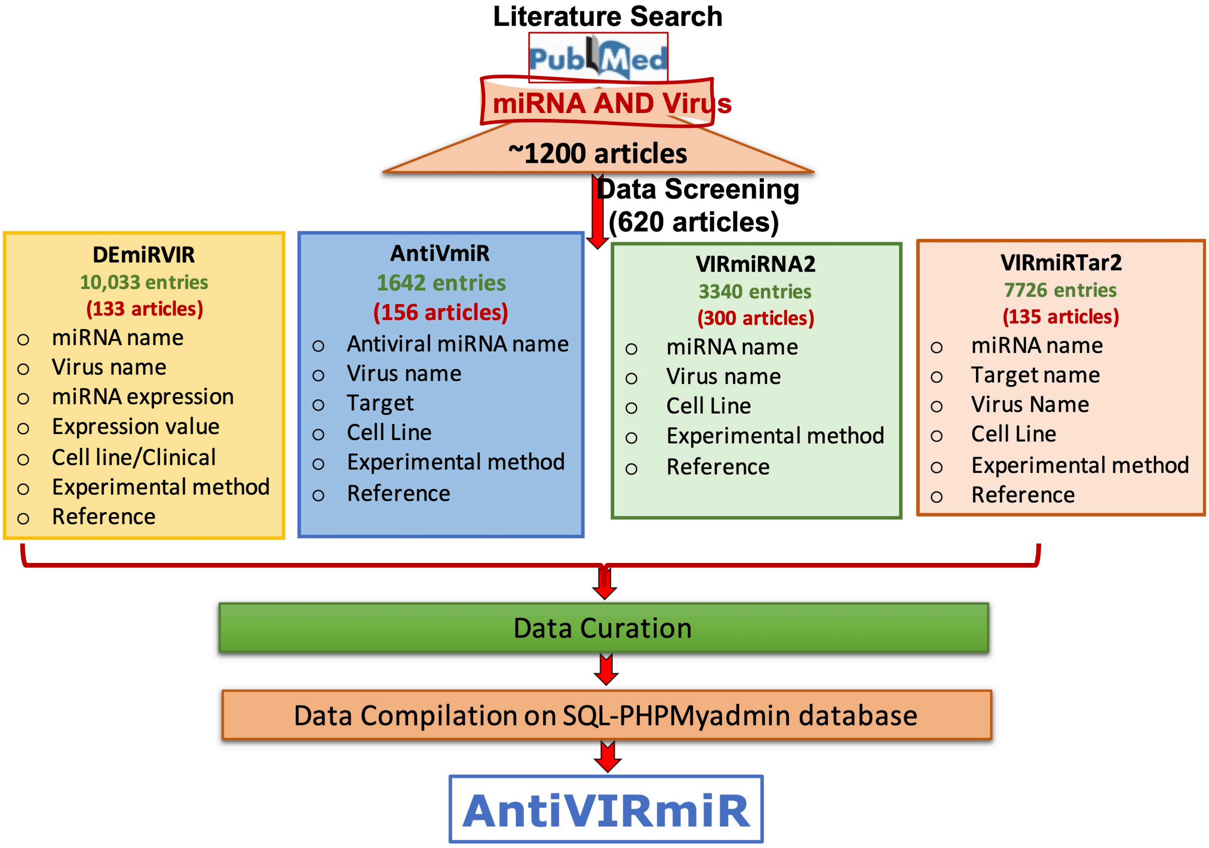

Supplementary Figure 1: Data generation flow of the AntiVIRmiR database

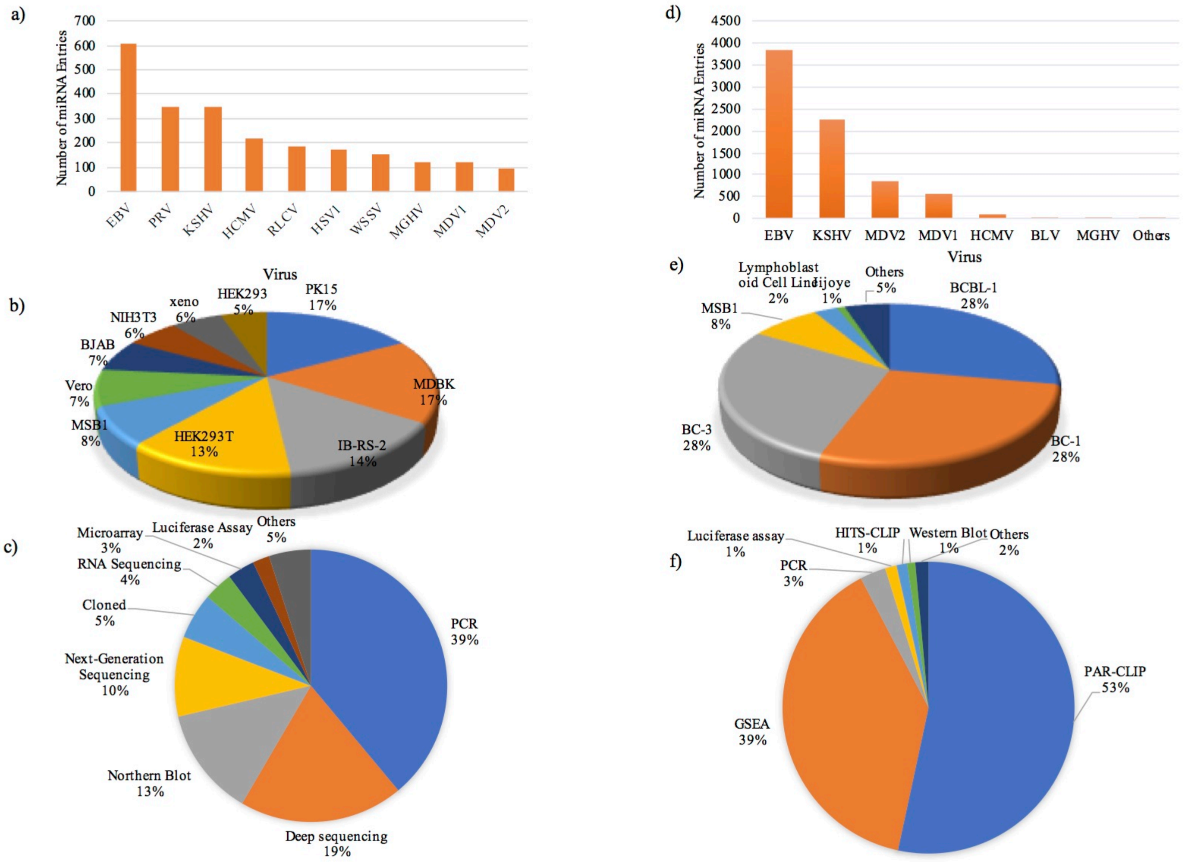

Supplementary Figure [illegible]. Data statistics: Bar graph represents number of entries in top 10 viruses for a) *'VIRmiRNA2'* and d) *'VIRmiRTar2'*. Pie-charts representing statistical distribution of cell lines in (b) *'VIRmiRNA2'* and (e) *'VIRmiRTar2'* and experimental methods in (c) *'VIRmiRNA2'* and (f) *'VIRmiRTar2'*. (EBV, Epstein Barr virus; PRV, Pseudorabies virus; KSHV, Kaposi sarcoma-associated herpesvirus; HCMV, Human cytomegalovirus; RLCV, Rhesus lymphocryptovirus; HSV1, Herpes simplex virus 1; WSSV, White Spot Syndrome virus; MGHV, Mouse gammaherpesvirus 68; MDV1, Marek's disease virus type 1; MDV2, Marek's disease virus type 2 and BLV, Bovine Leukemia Virus)

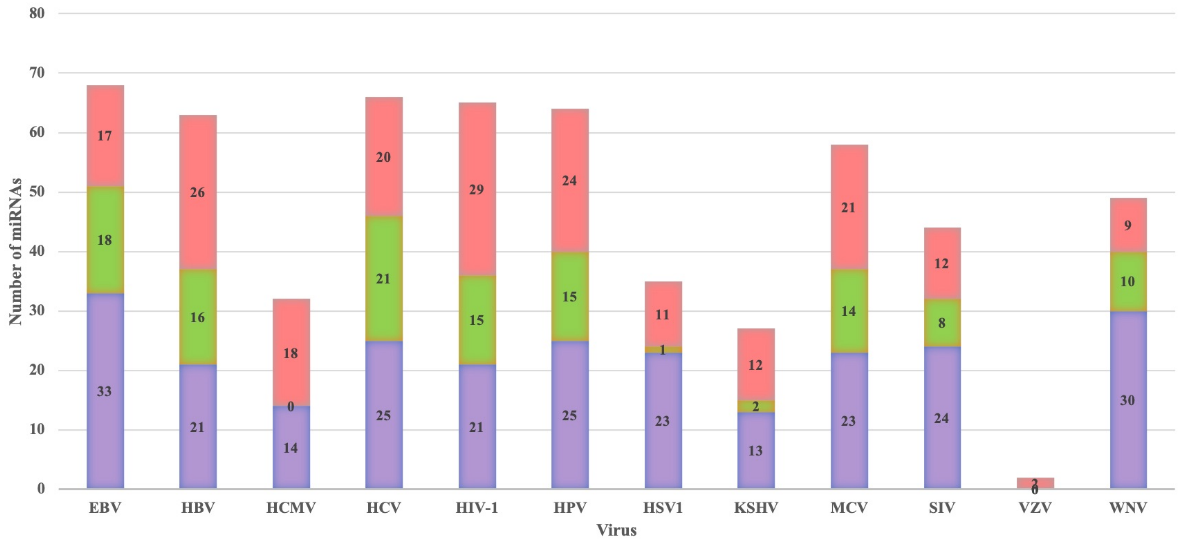

Supplementary Figure 3: *'DEmiRVIR'* statistics: Bar graph depicting the number of miRNAs that upregulate, downregulate or found in both the expression in viruses. Bar graph with purple color represents downregulation, red color represents upregulation and green color represents miRNAs found in both upregulation as well as downregulation. (EBV, Epstein Barr virus; HBV, Hepatitis B virus; HCMV, Human cytomegalovirus; HCV, Hepatitis C virus; HIV-1, Human immunodeficiency virus; HPV, Human papillomavirus; HSV1, Herpes simplex virus 1; KSHV, Kaposi sarcoma-associated herpesvirus; MCV, Merkel cell polyomavirus; SIV, Simian immunodeficiency virus; VZV, Varicella-zoster virus; WNV, West Nile Virus)

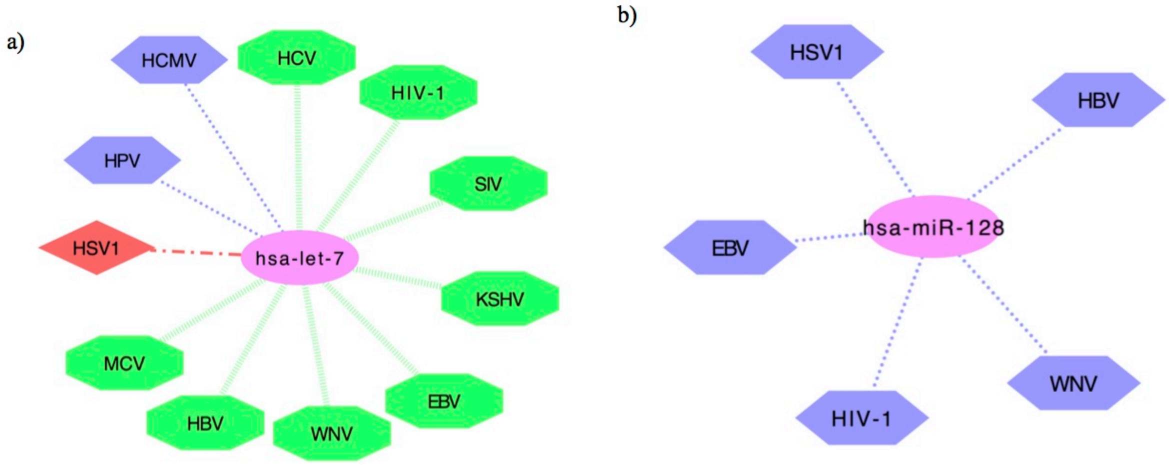

Supplementary Figure 4: Network showing human miRNA expression reported in different viruses in *'DEmiRVIR'* a) hsa-let-7 and b) hsa-miR-128. (Viruses with hexagon shape (purple

color) represent downregulation, diamond shape (red color) represents upregulation, octagon shape (green color) represent viruses found common in both upregulation as well as downregulation in different studies and miRNA is represented in ellipse shape (pink color)).

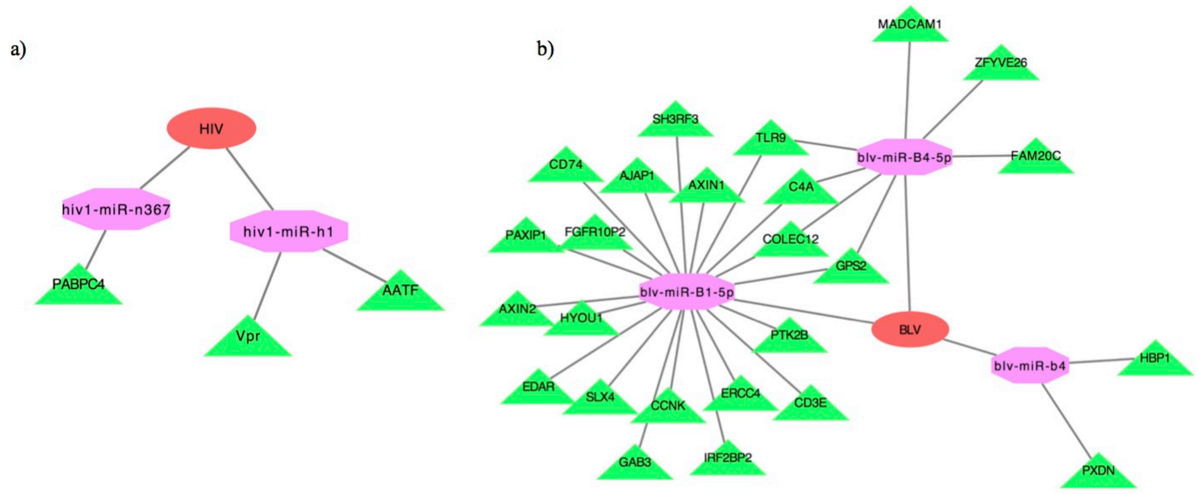

Supplementary Figure [illegible] Network interaction analysis showing viral miRNA target interaction analysis for **a)** HIV and **b)** BLV. (Virus is represented in ellipse shape (red color), miRNA in octagon shape (pink color) and targets in triangular shape (green color)).

Supplementary Table [illegible] **Table showing isomeric miRNA sequences for different viral miRNAs**

1. **Bovine foamy virus (BFV)**

S. No.	miRNA	miRNA Sequence	Length	PMID
1.	bfv-miR-bf1-3p	**ucccugaagccauauccgaggc**	22	24522910
		ucccugaagccauauccgaggcu	23	24522910
		ucccugaagccauauccgaggca	23	24522910
		ucccugaagccauauccgaggu	22	24522910
		ucccugaagccauauccgagg	21	24522910
2.	bfv-miR-bf1-5p	**uucggaggauggcucaucaagc**	22	24522910

		uucggaggauggcucaucaagcu	23	24522910
		uucggaggauggcucaucaagcc	23	24522910
		uucggaggauggcucaucaag	21	24522910
3.	bfv-miR-bf2-3p	**ucaggcgguaugcuuucuacuuu**	23	24522910
		ccaggcgguaugcuuucuacuuuu	24	24522910
		ccaggcgguaugcuuucuacuu	22	24522910
		ccaggcgguaugcuuucuacu	21	24522910
		ucaggcgguaugcuuucuacu	21	24522910
		ccaggcgguaugcuuucuac	20	24522910
4.	bfv-miR-bf2-5p	**ucaguagaaagacaguaccucgccu**	25	24522910
		ucaguagaaagacaguaccucgccugu	27	24522910
		ucaguagaaagacaguaccucgccc	25	24522910
		ucaguagaaagacaguaccucgcc	24	24522910
		ucaguagaaagacaguaccucgc	23	24522910

2. Bovine leukemia virus (BLV)

S. No.	miRNA	miRNA Sequence	Length	PMID
1.	blv-miR-b2-3p	**ugcgugucgcucagucauuuu**	21	22308400
		ugcgugucacucagucauuuu	21	22308400
2.	blv-miR-b4-3p	**uagcaccacagucucugcgccuuu**	24	22308400
		uagcaccauagucucugcgccuuu	24	22308400
		uagcaccaccgucucugcgccuuu	24	22308400
3.	blv-miR-b5-3p	**cucgagccgcaaccucccuuucu**	23	22308400
		cucaggccgcaaccucccuuucu	23	22308400
4.	blv-miR-b5-5p	**aggaagguuguggcucagaggu**	22	22308400
		aggaggguuguggcucagaggu	22	22308400

3. Duck enteritis virus (DEV)

S. No.	miRNA	miRNA Sequence	Length	PMID
1	dev-miR-d11-3p	**gcaaaagggcagccugggcucuau**	24	22492913
		aaaagggcagccugggcu	18	29704894
2	dev-miR-d12-3p	**cuccgcggugaggucccagaaa**	22	22492913
		cuccgcggugaggucccagaa	21	29704894
3	dev-miR-d13-5p	**cccgugggguagaacgcau**	19	22492913
		cguggguagaacgcaug	18	29704894
4	dev-miR-d14-3p	**gcguuaugucugguuauuauguuuuu**	26	22492913
		guuaugucugguuauuauguuuu	23	29704894
5	dev-miR-d15-3p	**cgagcguggggcaagguaccag**	21	22492913
		cgagcguggggcaagguacc	19	29704894
6	dev-miR-d17-3p	**gaccgcucgccuucgaggccacc**	23	22492913
		uccgaccgcucgccuucgaggc	22	29704894
7	dev-miR-d17-5p	**ugcaacgaaggcgaacgguug**	21	22492913
		ugcaacgaaggcgaacgguuga	22	29704894
8	dev-miR-d19-5p	**gaugaaagagcggugccuuu**	20	22492913
		augaaagagcggugccuuu	19	29704894
9	dev-miR-d21-3p	**auccaugcaaucuccaaacaacc**	23	22492913
		auccaugcaaucuccaaacaac	22	29704894
10	dev-miR-d21-5p	**ugguuuggagacagcugcgguggu**	24	22492913
		gguuuggagacagcugcggugg	22	29704894
11	dev-miR-d23-3p	**cgcgaaccgucacagucugcag**	22	22492913
		cgaaccgucacagucugcaga	21	29704894
12	dev-miR-d4-3p	**uuaauugucggauugguaugcuuuuu**	26	22492913
		uugucggauugguaugcuuu	20	29704894

13	dev-miR-d6-3p	**gucagagugucggugagucgacg**	23	22492913
		gucagagugucggugagucga	21	29704894
14	dev-miR-d7-5p	**uucguagcggcguauaaugguuu**	23	22492913
		cguagcggcguauaaugguuu	21	29704894
15	dev-miR-d8-3p	**uacaguuucguugggcgguuuc**	22	22492913
		uacaguuucguugggcgguuu	21	29704894
16	dev-miR-d8-5p	**ugccucccgauuaaacuauacgc**	23	22492913
		ugccucccgauuaaacuauacg	22	29704894
17	dev-miR-d9-3p	**caguccagaauguucaaacg**	20	22492913
		caguccagaauguucaaac	19	29704894
18	dev-miR-d9-5p	**cguuugaacguucuguacugccc**	23	22492913
		cguuugaacguucuguacugcc	22	29704894

4. Epstein Barr virus (EBV)

S. No.	miRNA	miRNA Sequence	Length	PMID
1.	ebv-miR-bart7-3p	**caucauaguccaguguccaggg**	22	16557291,17604727, 16540699
		aucauaguccaguguccagg	20	29425228
2.	ebv-miR-bart1-5p	**ucuuaguggaagugacgugcugug**	24	15118162, 17604727, 20808852
	ebv-miR-bart1-5p	cuuaguggaagugacgugcug	21	29425228
3.	ebv-miR-bart10-3p	**uacauaaccauggaguuggcugu**	23	16557291, 17604727, 16540699
	ebv-miR-bart10-3p	uacauaaccauggaguuggcu	21	29425228
4.	ebv-miR-bart12-5p	**acccgcccaucaccaccggaca**	22	24490137

		gacccgcccaucaccaccgga	21	24490137
		acccgcccaucaccaccggac	21	24490137
		acccgcccaucaccaccgga	20	24490137
		acccgcccaucaccaccggacu	22	24490137
5.	ebv-mir-bart16-3p	**uuagauagagugggugugugcucu**	24	16540699
		aucaccacccucuauccauau	21	24490137
		agaucaccacccucuauccaua	22	24490137
		agaucaccacccucuauccau	21	24490137
		agaucaccacccucuauccauau	23	24490137
		gaucaccacccucuauccauau	22	24490137
6.	ebv-miR-bart17-5p	**uaagaggacgcaggcauacaag**	22	16540699, 17604727, 30615681
		uaagaggacgcaggcauaca	20	29425228
7.	ebv-miR-bart19-3p	**uuuuguuugcuugggaaugcu**	21	16540699, 17604727, 30548946
		agaggacgcaggcauaca	18	29425228
		uuuuguuugcuugggaaugc	20	29425228
8.	ebv-miR-bart2-5p	**uauuuucugcauucgcccuugc**	22	15118162, 17604727, 20808852
		uauuuucugcauucgcccuug	21	29425228
9.	ebv-miR-bart22-3p	**uuacaaagucauggucuaguagu**	23	29425228
		uacaaagucauggucuaguagu	22	29425228
10.	ebv-miR-bart22-5p	**ugcuagacccuggaguugaacc**	22	24490137
		ugcuagacccuggaguugaac	21	24490137

		ugcuagacccuggaguug	18	24490137
		ugcuagacccuggaguugaaccu	23	24490137
		ugcuagacccuggaguu	17	24490137
		ugcuagacccuggaguugaaca	22	24490137
11.	ebv-miR-bart7*	ccuggaccuugacuaugaaaca	22	16557291
		**ccuggaccuugacuaugaaac**	21	26401047, 27956246
12.	ebv-miR-bart8-3p	**gucacaaucuauggggucguaga**	23	27147748, 30477559, 30548946, 30411781, 27239439, 32714979, 29230817, 31471531, 31938442
		gucacaaucuauggggucguag	22	29425228
		ucacaaucuauggggucguag	21	29425228
13.	ebv-miR-bhrf1-1-5p	**uaaccugaucagccccggaguu**	22	24490137, 30615681
		aaccugaucagccccggaguu	21	24490137
14.	ebv-miR-bhrf1-2-3p	**uaucuuuugcggcagaaauuga**	22	24490137, 32714979, 27239439, 20808852, 15118162
		uaucuuuugcggcagaaauug	21	24490137

15.	ebv-miR-bhrf1-2-5p	**aaauucuguugcagcagauagc**	22	32714979, 27239439, 31856276, 30615681
		aaauucuguugcagcagauag	21	24490137

5. Herpes B virus (HBV)

S. No.	miRNA	miRNA Sequence	Length	PMID
1.	hbv-miR-b14rc-3p	**aggaggggucugggagagaaggg**	23	21543500
		aggaggggucugggagagaagg	22	21543500
		ggaggggucugggagagaaggg	22	21543500
		aggaggggucugggagagaag	21	21543500
		ggaggggucugggagagaa	19	21543500
2.	hbv-miR-b19-5p	**gugggcgcacucgaucccggcu**	22	21543500
		gugggcgcacucgaucccggcugu	24	21543500
		gugggcgcacucgaucccggcug	23	21543500
		gugggcgcacucgaucccggc	21	21543500
		ggugggcgcacucgaucc	18	21543500
3.	hbv-miR-b2-3p	**cggccgggaacgagagacggcgg**	23	21543500
		cgggaacgagagacggcgg	19	21543500
		ccgggaacgagagacggc	18	21543500
4.	hbv-miR-b20-3p	**gugcugguugcccgcgcucucg**	22	21543500
		ugcugguugcccgcgcucucggg	23	21543500
		cgugcugguugcccgcgcucucg	23	21543500
		ugcugguugcccgcgcucucgg	22	21543500

		ugcugguugcccgcgcucucg	21	21543500
5.	hbv-miR-b20-5p	**agagugagggguggcgagcgcgu**	22	21543500, 19144716
		agugaggguggcgagcgcgugga	23	21543500
		agugaggguggcgagcgcgugg	22	21543500
		agagugaggguggcgagcgcg	21	21543500
		agagugaggguggcgagcgc	20	21543500
		agagugaggguggcgagcg	19	21543500
6.	hbv-miR-b21rc-5p	**gcgccggggugcgaaccugggg**	22	21543500
		gcgccggggugcgaaccuggggc	23	21543500
		agcgccggggugcgaaccugggg	23	21543500
		agcgccggggugcgaaccuggg	22	21543500
		cgccggggugcgaaccugggg	21	21543500
		gcgccggggugcgaaccugg	20	21543500
7.	hbv-miR-b22-3p	**uaggggaguccgugagagagcgg**	23	21543500
		uaggggaguccgugagagagcg	22	21543500
8.	hbv-miR-b26-5p	**ugaguucgggcagcaggcgcgu**	22	21543500
		ugaguucgggcagcaggcgcg	21	21543500
9.	hbv-miR-b3rc-3p	**cggcucugcguuuauuguuggaa**	23	21543500
		ggucucggcucugcguuuauugu	23	21543500
		cucggcucugcguuuauugu	20	21543500
		gucucggcucugcguuua	18	21543500
10.	hbv-miR-b7-5p	**uucugggccucggguucgcuuc**	22	21543500
		uucugggccucggguucgcuuccg	24	21543500
		cuucugggccucggguucgcuucc	24	21543500

		uucugggccucggguucgcuucc	23	21543500
		cuucugggccucggguucgcuuc	23	21543500
		cuucugggccucggguucgcuu	22	21543500
		ucugggccucggguucgcuucc	22	21543500
		uucugggccucggguucgcuu	21	21543500
		cuucugggccucggguucgcu	21	21543500
		ucugggccucggguucgcuuc	21	21543500
		ucugggccucggguucgcuu	20	21543500
11.	hbv-miR-b8-5p	**uguggaggccuaggggagcccgg**	23	21543500
		uguggaggccuaggggagcccggc	24	21543500
		gaucuucacuaggccugcccggcc	24	21543500
		cgaucuucacuaggccugcccggc	24	21543500
		cggcuguggaggccuaggggagc	23	21543500
		cgaucuucacuaggccugcccgg	23	21543500
		uguggaggccuaggggagcccg	22	21543500
		gaucuucacuaggccugcccgg	22	21543500
		aucuucacuaggccugcccggc	22	21543500
		uguggaggccuaggggagccc	21	21543500
		gaucuucacuaggccugcccg	21	21543500
		aucuucacuaggccugcccgg	21	21543500
		ucuucacuaggccugcccggc	21	21543500
		uguggaggccuaggggagcc	20	21543500
		gaucuucacuaggccugccc	20	21543500
		ucuucacuaggccugcccgg	20	21543500
		uguggaggccuaggggagc	19	21543500
		uucacuaggccugcccggc	19	21543500

		cuucacuaggccugcccgg	19	21543500

6. Human cytomegalovirus (HCMV)

S. No.	miRNA	miRNA Sequence	Length	PMID
1.	hcmv-miR-UL148D	**ucguccucccuucuucaccg**	21	31749099, 15782219, 22013051
		ucguccuccccuucuucaccu	21	31749099
2.	hcmv-miR-UL112-3p	**aagugacggugagauccaggcu**	22	31749099, 25955717, 28589873
		aagugacggugagauccaggc	21	31749099
3.	hcmv-miR-UL112-5p	**ccuccggaucacaugguuacuca**	23	31749099, 26067606, 27491954
		ccuccggaucacaugguuacucag	24	31749099
4.	hcmv-miR-UL22A-5p	**uaacuagccuucccgugaga**	20	31749099
		cuaacuagccuucccgugaga	21	31749099, 24533100
5.	hcmv-miR-UL36-5p	**ucguugaagacaccuggaaaga**	22	31749099, 24533100, 28589873
		ucguugaagacaccuggaaag	21	31749099
6.	hcmv-miR-UL36-3p	**uuuccagguguuuucaacgugc**	22	24533100, 28589873
		uuuccagguguuuucaacgug	21	31749099, 26067606

7.	hcmv-miR-UL59	**guucucucgcucgucaugccgu**	22	27491954, 28592251
		uucucucgcucgucaugccg	20	31749099
		uucucucgcucgucaugcc	19	31749099
8.	hcmv-miR-UL70-5p	**ugcgucucggccucguccaga**	21	16140786, 22715351, 24533100
		cgucucggccucguccagac	20	31749099
9.	hcmv-miR-US25-1-3p	**uccgaacgcuaggucgguucu**	21	31749099, 24533100
		guccgaacgcuaggucgguucu	22	31749099
		uccgaacgcuaggucgguucuc	22	28589873
10.	hcmv-miR-US25-1-5p	**aaccgcucaguggcucggacc**	21	31749099, 24533100, 28589873
		aaccgcucaguggcucggaccg	22	31749099
11.	hcmv-miR-US25-2-3p	**auccacuuggagagcucccgcggu**	24	31749099, 25955717, 24533100
		auccacuuggagagcucccgcgg	23	15782219, 22013051, 22715351
12.	hcmv-miR-US25-2-5p	**agcggucuguucagguggauga**	22	15782219, 22013051, 25955717
		agcggucuguucagguggaug	21	31749099
13.	hcmv-miR-US29-3p	**cccacgguccgggcacaauca**	21	26067606,

				28592251
		cccacgguccgggcacaaucaac	23	31749099
14.	hcmv-miR-US33-3p	**ucacgguccgagcacauccaa**	21	24533100, 26067606, 27491954
		ucacgguccgagcacaucca	20	15782219, 22013051, 22715351
15.	hcmv-miR-US33-5p	**gauugugcccggaccgugggcg**	22	15782219, 22013051, 22715351
		auugugcccggaccgugggcgc	22	31749099

7. Herpes simplex virus 1 (HSV1)

S. No.	miRNA	miRNA Sequence	Length	PMID
1.	hsv1-miR-H2-3p	**ccugagccagggacgagugcgacu**	24	21795359, 25535379, 32390978
		cugagccagggacgagugcga	21	19656888
		cugagccagggacgagugcgacu	23	19656888
		ugagccagggacgagugcgacu	22	19656888
2.	hsv1-miR-H3-3p	**cugggacugugcgguugggac**	21	19656888, 25535379, 32390978
		cugggacugugcgguugg	18	19656888
		cugggacugugcgguuggg	19	19656888
		cugggacugugcgguuggga	20	19656888

3.	hsv1-miR-H5-3p	**gucagagauccaaacccuccgg**	22	21795359
		cagagauccaaacccuccg	19	19656888
4.	hsv1-miR-H6-3p	**cacuucccguccuuccauccc**	21	19656888, 25535379, 31020575
		ccacuucccguccuuccauccc	22	19656888
		cacuucccguccuuccaucc	20	19656888
5.	hsv1-miR-H7-5p	**aaaggggucugcaaccaaagg**	21	19656888, 25535379
		gaaaggggucugcaaccaaagg	22	19656888

8. Kaposi sarcoma-associated herpesvirus (KSHV)

ID	miRNA	miRNA Sequence	Length	PMID
1.	kshv-miR-K12-11-3p	**uuaaugcuuagccuguguccga**	22	27611973, 30533200
		uuaaugcuuagccuguguccg	21	29425228
		uaaugcuuagccuguguccga	21	29425228
		augcuuagccuguguccg	18	29425228
		ccuuaaugcuuagccuguguccg	23	29425228
2.	kshv-miR-k12-12-3p	**ugggggagggugcccugguuga**	22	27611973, 28600495
		ugggggagggugcccugguug	21	29425228
		gggagggugcccugguugac	20	29425228
3.	kshv-miR-K12-12-5p	**aaccaggccaccauucccucuccg**	23	29425228, 27611973
		cuaaccaggccaccauucccucuc	23	29425228
		ugucaaccaggccaccauu	19	29425228

4.	kshv-miR-K12-2-5p	**aacuguaguccgggucgaucug**	22	27611973, 28165144
		aacuguaguccgggucgau	19	29425228
		acuguaguccgggucgau	18	29425228
5.	kshv-miR-K12-4-3p	**uagaauacugaggccuagcuga**	22	24027049, 29846699
		uagaauacugaggccuagcug	21	29425228
		agaauacugaggccuagcug	20	29425228
6.	kshv-miR-K12-7-5p	**agcgccaccggacggggauuuaug**	24	29846699, 28165144
		ugagcgccaccggacgggg	19	29425228
7.	kshv-miR- K12-8-3p	**cuaggcgcgacugagagagca**	21	15800047
		cuaggcgcgacugagagag	19	15800047
		uaggcgcgacugagagagcacg	22	27611973, 30533200
		cuaggcgcgacugagagagc	20	29425228
		uaggcgcgacugagagagc	19	29425228
8.	kshv-miR-K12-8-5p	**acucccucacuaacgccccgcu**	22	29425228, 27611973
		cucccucacuaacgccccgcu	21	29425228
		cacucccucacuaacgcccc	20	29425228
		gcacucccucacuaacgcccc	21	29425228
9.	kshv-miR-K12-9-3p	**cuggguauacgcagcugcguaa**	22	28600495, 27611973
		cuggguauacgcagcugcgu	20	29425228
10.	kshv-miR-K12-9-5p	**acccagcugcguaaaccccgcu**	22	27611973, 30533200,

				28165144
		acccagcugcguaaaccccg	20	29425228
		uacccagcugcguaaacccc	20	29425228
11.	kshv-miR-K4-5p	**agcuaaaccgcaguacucuagg**	22	15800047, 27611973, 28102325
		agcuaaaccgcaguacucuag	21	15800047
12.	kshv-miR-K12-7-3p	**ugaucccauguugcuggcgc**	20	29846699
		ugaucccauguugcuggcgcu	21	15800047
		ugaucccauguugcuggcgcuca	23	15800047

9. Marek's disease virus 1 (MDV1)

S. No.	miRNA	miRNA Sequence	Length	PMID
1.	mdv1-miR-m1/ mdv1-m1-5p	**ugcuuguucacugugcggca**	20	16912324, 18842708, 21645299, 24449754
		ugcuuguucacugugcggcauu	22	24449754
		ugcuuguucacugugcggcauua	23	24449754
2.	mdv1-m11-5p	**uuuuccuuaccguguagcuuaga**	23	24449754
		uuuuccuuaccguguagcuuag	22	24449754
3.	mdv1-miR-m12-3p/ mdv1-m12-3p	**ugcauaauacggaggguucu**	20	21645299, 24449754, 30184155
		uugcauaauacggaggguucug	22	29423087
		uugcauaauacggaggguucugu	23	24449754
		ugcauaauacggaggguucugu	22	24449754

4.	mdv1-miR-m2-3p	**cggacugccgcagaauagcuu**	21	16912324, 18256158, 18842708, 24449754
		cagacccccucucccccucuuu	22	29767614
		acggacugccgcagaauagcuuu	23	29423087
5.	mdv1-m2-5p	**guuguauucugcccgguaguccg**	23	24449754
		guuguauucugcccgguaguccgu	24	24449754
		guuguauucugcccgguaguccguu	25	24449754
6.	mdv1-m3-5p/ mdv1-miR-m3-5p	**augaaaaugugaaaccucucccgc**	24	24449754
		ugaaaaugugaaaccucucccgc	23	24449754
		caugaaaaugugaaaccucucccgcu	26	29423087
7.	mdv1-m31-3p	**ugcuacagucgugagcagaucaa**	23	24449754
		ugcuacagucgugagcagauc	21	24449754
		ugcuacagucgugagcagauca	22	24449754
8.	mdv1-m4-5p	**uuaaugcuguaucggaacccuuc**	23	24449754
		uuaaugcuguaucggaacccuucgu	25	24449754
		uuaaugcuguaucggaacccuucg	24	24449754
9.	mdv1-miR-m5-3p	**uguguaucguggucgucuacugu**	23	21645299, 24449754, 30184155
		uguguaucguggucgucuacuguu	24	29423087
9.	mdv1-miR-m6-5p/ mdv1-m6-5p	**ucuguuguuccguaguguucuc**	22	16912324, 18256158, 18842708,

				24449754
		uguuguuccguaguguucucg	21	24449754
		uguuguuccguaguguucucgu	22	24449754
10.	mdv1-miR-m7*/ mdv1-m7-5p	**uguuaucucggggagaucccgau**	23	16912324
		guuaucucggggagaucucgau	22	24449754
		uguuaucucggggagaucucga	22	24449754
		guuaucucggggagaucucga	21	24449754
11.	mdv1-m8-3p	**gugaccucuacggaacaauagu**	22	16912324
		ugaccucuacggaacaauagu	21	24449754
		ugaccucuacggaacaauagc	21	24449754
12.	mdv1-miR-m9-5p	**uuuucuccuucccccggaguu**	22	18256158, 18842708, 21645299, 24449754, 30184155
		uuuucuccuucccccggaguuca	24	29423087

10. Marek's disease virus 2 (MDV2)

S. No.	miRNA	miRNA Sequence	Length	PMID
1.	mdv2-m14-5p	**ugugguacggugcacccugaga**	22	24449754
		gugugguacggugcacccugaga	23	24449754
2.	mdv2-m18-5p	**uguuuucucucaggcuggcauug**	23	17459919
		guuuucucucaggcuggcauugc	23	24449754
		guuuucucucaggcuggcauugca	24	24449754
		uguuuucucucaggcuggcauugc	24	24449754
3.	mdv2-m19-5p	**ccccucggcggugugcacgg**	20	24449754
		ccccucggcggugugcacggg	21	24449754

		ccccucggcggugugcacgggu	22	24449754
		ccccucggcggugugcacggga	22	24449754
4.	mdv2-m20-5p	**uccuuagcguggugccugaga**	21	24449754
		uccuuagcguggugccugag	20	24449754
5.	mdv2-m22-5p	**ucuuacacgcacgucacucuggu**	23	24449754
		ucuuacacgcacgucacucugguc	24	24449754
6.	mdv2-m24-3p	**uuagaugccgucagggaaagau**	22	24449754
		uagaugccgucagggaaagaug	22	24449754
7.	mdv2-m25-5p	**ccuccuucggacgagugcuugcc**	23	24449754
		ccuccuucggacgagugcuugc	22	24449754
		ccuccuucggacgagugcuugccg	24	24449754
		uccuucggacgagugcuugccg	22	24449754
8.	mdv2-m26-3p	**ucgggcaccgcaccgaaggaug**	22	24449754
		ucgggcaccgcaccgaaggaugu	23	24449754
		ucgggcaccgcaccgaaggau	21	24449754
		ucgggcaccgcaccgaaggauu	22	24449754
9.	mdv2-miR-m27-3p/mdv2-m27-3p	**gcgucgagcaccgugcuggagga**	23	17459919
		cgucgagcaccgugcuggagga	22	24449754
		cgucgagcaccgugcuggaggaa	23	24449754
10.	mdv2-m27-5p	**cuucguccgguguucgaggcg**	21	24449754
		cuucguccgguguucgaggcgu	22	24449754
11.	mdv2-m28-5p	**uuuucucgacgccuacccucgg**	22	24449754
		uucucgacgccuacccucggc	21	24449754
		uuuucucgacgccuacccucggc	23	24449754
		uucucgacgccuacccucggcg	22	24449754
12.	mdv2-m30-5p	**caacacucccucggacgcagca**	22	24449754
		caacacucccucggacgcagc	21	24449754

11. Pseudorabies virus (PRV)

S. No.	miRNA	miRNA Sequence	Length	PMID
1.	prv-miR-1-3p	ucucaccccugggucgucgc	21	22292087
		ucucaccccugggucgucgcc	22	22292087
		cucucaccccugggucgucgc	22	22292087
		cucucaccccugggucgucg	21	22292087
		ucucaccccugggucgucg	20	22292087
		ucucaccccugggucguc	19	22292087
2.	prv-miR-1-5p	gacggcuccuggggcugaaagcggcgc	27	22292087
		gacggcuccuggggcugaaagcgg	24	22292087
		gacggcuccuggggcugaaagcg	23	22292087
		acggcuccuggggcugaaagcgg	23	22292087
		gacggcuccuggggcugaaagc	22	22292087
		gacggcuccuggggcugaaag	21	22292087
		ggcggucggggggcgcgucgggc	23	26998839
3.	prv-miR-10-1	ccgagccugccccuuccgucgca	23	22292087
		ccgagccugccccuuccgucgcac	24	22292087
		cgagccugccccuuccgucgcac	23	22292087
		cugccccuuccgucgcaccgggg	23	22292087
		uccgcggaucgcaucggcgcgc	22	22292087
		ccgagccugccccuuccgucgc	22	22292087
		cgagccugccccuuccgucgca	22	22292087
		gagccugccccuuccgucgcac	22	22292087
		ccgagccugccccuuccgucg	21	22292087
		cgagccugccccuuccgucgc	21	22292087
		ccgagccugccccuuccguc	20	22292087

		cgagccugccccuuccgucg	20	22292087
		agccugccccuuccgucgca	20	22292087
		ccgagccugccccuuccgu	19	22292087
		cgagccugccccuuccguc	19	22292087
		gagccugccccuuccgucg	19	22292087
		ccgagccugccccuuccg	18	22292087
		gagccugccccuuccguc	18	22292087
		agccugccccuuccgucg	18	22292087
4.	prv-miR-11-1	aggcugggaguggggacggaaga	23	22292087
		aggcugggaguggggacggaagacgg	26	22292087
		guggggacggaagacggaagccagau	26	22292087
		uuccgcccgcucucccaccgccuuuc	26	22292087
		aggcugggaguggggacggaagacg	25	22292087
		gcugggaguggggacggaagacgga	25	22292087
		ggggacggaagacggaagccagaug	25	22292087
		uuccgcccgcucucccaccgccuuu	25	22292087
		aaccuguuccgcccgcucucccacc	25	22292087
		gaggcugggaguggggacggaaga	24	22292087
		aggcugggaguggggacggaagac	24	22292087
		guggggacggaagacggaagccag	24	22292087
		cugggaguggggacggaagacgg	23	22292087
		gggacggaagacggaagccagau	23	22292087
		ggacggaagacggaagccagaug	23	22292087
		guuccgcccgcucucccaccgcc	23	22292087
		agaugaaccuguuccgcccgcuc	23	22292087
		ugaaccuguuccgcccgcucucc	23	22292087
		aggcugggaguggggacggaag	22	22292087

		ggcugggaguggggacggaaga	22	22292087
		gcugggaguggggacggaagac	22	22292087
		cugggaguggggacggaagacg	22	22292087
		gggacggaagacggaagccaga	22	22292087
		gacggaagacggaagccagaug	22	22292087
		cggaagacggaagccagaugaa	22	22292087
		agaugaaccuguuccgcccgcu	22	22292087
		aggcugggaguggggacggaa	21	22292087
		ggcugggaguggggacggaag	21	22292087
		gcugggaguggggacggaaga	21	22292087
		gggacggaagacggaagccag	21	22292087
		cggaagacggaagccagauga	21	22292087
		gggaggcugggagugggga	20	22292087
		aggcugggaguggggacgga	20	22292087
		gcugggaguggggacggaag	20	22292087
		gggaguggggacggaagacg	20	22292087
		accuguuccgcccgcucucc	20	22292087
		aggcugggaguggggacgg	19	22292087
		cugggaguggggacggaag	19	22292087
		acggaagacggaagccaga	19	22292087
		aggcugggaguggggacg	18	22292087
		aagacggaagccagauga	18	22292087
5.	prv-miR-2-3p	ucgaggacgggcgcggggcaac	22	22292087
		cgcggggcaacgguggugagag	22	22292087
		cgcggggcaacgguggugaga	21	22292087
		cgcggggcaacgguggugag	20	22292087
		cgcggggcaacggugguga	19	22292087

		cggggcaacgguggugag	18	22292087
		caugcaccuguaccucucgg	20	26998839
6.	prv-miR-2-5p	cucaucccgucagaccugcg	20	22292087
		cucaucccgucagaccugcgccau	24	22292087
		gcuucucaucccgucagaccugcg	24	22292087
		uucucaucccgucagaccugcgcc	24	22292087
		cucaucccgucagaccugcgcca	23	22292087
		cucaucccgucagaccugcgcc	22	22292087
		cauucgcuucucaucccgucag	22	22292087
		ucucaucccgucagaccugcg	21	22292087
		cucaucccgucagaccugcgc	21	22292087
		ucaucccgucagaccugcgcc	21	22292087
		cccgucagaccugcgccaug	20	22292087
		cauucgcuucucaucccguc	20	22292087
		cucaucccgucagaccugc	19	22292087
		ucucaucccgucagaccu	18	22292087
		cucaucccgucagaccug	18	22292087
7.	prv-miR-3-3p	cgcacacgccccucucgcgcac	22	22292087
		cgcacacgccccucucgcgca	21	22292087
		gcacacgccccucucgcgcac	21	22292087
		gcacacgccccucucgcgca	20	22292087
		cggccagcccggacgcgcugua	22	26998839
8.	prv-miR-4-3p	aaaaggcacgcugaugcgucc	21	22292087
		cgacgacugggggcgcgcgcc	21	26998839
9.	prv-miR-4-5p	agaguaucagcguggcuuuuuu	22	22292087
		cuuuuuuguuggucagggugugaaaaag	28	22292087
		aagaguaucagcguggcuuuuuug	24	22292087

		caagaguaucagcguggcuuuuu	23	22292087
		aagaguaucagcguggcuuuuuu	23	22292087
		agaguaucagcguggcuuuuuug	23	22292087
		caagaguaucagcguggcuuuu	22	22292087
		aagaguaucagcguggcuuuuu	22	22292087
		ucagcguggcuuuuuuguuggu	22	22292087
		caagaguaucagcguggcuuu	21	22292087
		aagaguaucagcguggcuuuu	21	22292087
		agaguaucagcguggcuuuuu	21	22292087
		gaguaucagcguggcuuuuuu	21	22292087
		aagaguaucagcguggcuuu	20	22292087
		agaguaucagcguggcuuuu	20	22292087
		gaguaucagcguggcuuuuu	20	22292087
		aguaucagcguggcuuuuuu	20	22292087
		caagaguaucagcguggcu	19	22292087
		aagaguaucagcguggcuu	19	22292087
		agaguaucagcguggcuuu	19	22292087
		gaguaucagcguggcuuuu	19	22292087
		aagaguaucagcguggcu	18	22292087
		agaguaucagcguggcuu	18	22292087
10.	prv-miR-5-3p	auucauccgugcgguggugggau	24	22292087
		cuguuccccauccuuccacccau	23	22292087
		cgagcucugcgaccggcgcg	20	26998839
11.	prv-miR-5-5p	uccgugcgguggugggaugagug	24	22292087
		gggaugaguggauggauggaggcg	24	22292087
		augaguggauggauggaggcgag	23	22292087
		ugaguggauggauggaggcgagg	23	22292087

		augaguggauggauggaggcga	22	22292087
		aguggauggauggaggcgaggg	22	22292087
		uggggaugaguggauggaugg	21	22292087
		augaguggauggauggaggcg	21	22292087
		ugaguggauggauggaggcga	21	22292087
		gaguggauggauggaggcgag	21	22292087
		uggggaugaguggauggaug	20	22292087
		augaguggauggauggaggc	20	22292087
		ugaguggauggauggaggcg	20	22292087
		aguggauggauggaggcgag	20	22292087
		gaugaguggauggauggag	19	22292087
		augaguggauggauggagg	19	22292087
		ugaguggauggauggaggc	19	22292087
		gaguggauggauggaggcg	19	22292087
		aguggauggauggaggcga	19	22292087
		guggauggauggaggcgag	19	22292087
		ggauggauggaggcgaggg	19	22292087
		uggggaugaguggaugga	18	22292087
		augaguggauggauggag	18	22292087
		ugaguggauggauggagg	18	22292087
		aguggauggauggaggcg	18	22292087
		uggauggauggaggcgag	18	22292087
12.	prv-miR-6-3p	cuuggcagcggguggguaccca	22	22292087
		cuuggcagcggguggguacc	20	22292087
		cuuggcagcggguggguac	19	22292087
		cuuggcagcggguggguа	18	22292087
13.	prv-miR-6-5p	cguaccgacccgccuaccagg	21	22292087

		cguaccgacccgccuaccaggca	23	22292087
		cguaccgacccgccuaccaggc	22	22292087
		cguaccgacccgccuaccag	20	22292087
		cguaccgacccgccuacca	19	22292087
		cguaccgacccgccuacc	18	22292087
		cgcaggcgcgcggcauggaggu	22	26998839
14.	prv-miR-7-3p	accaccgucccccugucccu	20	22292087
		ugucccucauccacuucucgacggccggc	29	22292087
		accaccgucccccugucccuc	21	22292087
		uccacuucucgacggccggcu	21	22292087
		accaccgucccccugucccc	19	22292087
		accaccgucccccugucc	18	22292087
15.	prv-miR-7-5p	gggaugggcgcucggggguga	21	22292087
		cgcucgggggugaucgucugcuacc	25	22292087
		cgcucgggggugaucgucugcuac	24	22292087
		cccgggggguugaugggggauggg	23	22292087
		cgcucgggggugaucgucugcua	23	22292087
		cccgggggguugauggggaugg	22	22292087
		ccgggggguugauggggauggg	22	22292087
		cucgggggugaucgucugcuac	22	22292087
		ucgggggugaucgucugcuacc	22	22292087
		ccgggggguugauggggaugg	21	22292087
		cgggggguugauggggauggg	21	22292087
		cccgggggguugauggggau	20	22292087
		ccgggggguugauggggaug	20	22292087
		cgggggguugauggggaugg	20	22292087
		gggaugggcgcucgggggug	20	22292087

		ccggggggguugauggggau	19	22292087
		cccgggggguugaugggga	19	22292087
		cgggggguugauggggaug	19	22292087
		gggaugggcgcucgggggu	19	22292087
		cccgggggguugaugggg	18	22292087
		ccgggggguugaugggga	18	22292087
		cgggggguugauggggau	18	22292087
16.	prv-miR-8-3p	caacccuucuggagcccuacc	21	22292087
		aacccuucuggagcccuacccucugugcc	29	22292087
		gguguucucaacccuucuggagcccu	26	22292087
		caacccuucuggagcccuacccuc	24	22292087
		caacccuucuggagcccuacccu	23	22292087
		aaccgguguucucaacccuucug	23	22292087
		caacccuucuggagcccuaccc	22	22292087
		accgguguucucaacccuucug	22	22292087
		ccuucuggagcccuacccucug	22	22292087
		caacccuucuggagcccuac	20	22292087
		aacccuucuggagcccuacc	20	22292087
		aaccgguguucucaacccuu	20	22292087
		caacccuucuggagcccua	19	22292087
		cgagcuccugccggcccgcacg	22	26998839
17.	prv-miR-8-5p	guggggcgaagauuggguu	20	22292087
		uggggcgaagauuggguugggugaga	27	22292087
		ggguugggugagagacuagaaccggug	27	22292087
		guggggcgaagauuggguugggu	24	22292087
		ggugagagacuagaaccgguguuc	24	22292087
		guggggcgaagauuggguuggg	23	22292087

		ggggcgaagauuggguuggguga	23	22292087
		ggugagagacuagaaccgguguu	23	22292087
		caggcgguggggcgaagauug	22	22292087
		guggggcgaagauuggguugg	22	22292087
		uggggcgaagauuggguuggg	22	22292087
		gggugagagacuagaaccggug	22	22292087
		ggugagagacuagaaccggugu	22	22292087
		gugagagacuagaaccgguguu	22	22292087
		cgguggggcgaagauugggu	21	22292087
		guggggcgaagauuggguug	21	22292087
		uggggcgaagauuggguugg	21	22292087
		gugagagacuagaaccggugu	21	22292087
		uggggcgaagauuggguug	20	22292087
		gcgaagauuggguugggugа	20	22292087
		gugagagacuagaaccggug	20	22292087
		guggggcgaagauugggu	19	22292087
		uggggcgaagauuggguu	19	22292087
		guggggcgaagauuggg	18	22292087
		uggggcgaagauugggu	18	22292087
		gggggcgaagauuggguu	18	22292087
		ggggcgaagauuggguug	18	22292087
		gggcgaagauuggguugg	18	22292087
		gugagagacuagaaccgg	18	22292087
18.	prv-miR-8-loop	ugagagacuagaaccgguguucu	23	22292087
		ugagagacuagaaccgguguuc	22	22292087
		gagagacuagaaccgguguucu	22	22292087
		uagaaccgguguucucaacccu	22	22292087

		ugagagacuagaaccgguguu	21	22292087
		ugagagacuagaaccggugu	20	22292087
		ugagagacuagaaccggug	19	22292087
		ugagagacuagaaccggu	18	22292087
19.	prv-miR-9-3p	ccucccccgcaucucuucucuc	22	22292087
		cccucccccgcaucucuucuc	21	22292087
		ccucccccgcaucucuucucu	21	22292087
		cccucccccgcaucucuucu	20	22292087
		ccucccccgcaucucuucuc	20	22292087
		cucccccgcaucucuucucu	20	22292087
		cccucccccgcaucucuuc	19	22292087
		ccucccccgcaucucuucu	19	22292087
		cucccccgcaucucuucuc	19	22292087
		cccucccccgcaucucuu	18	22292087
		ccucccccgcaucucuuc	18	22292087
		cucccccgcaucucuucu	18	22292087
		cccccgcaucucuucucu	18	22292087
20.	prv-miR-9-5p	aucgaggagauguggagggg	20	22292087
		gaggagauguggaggggugccaagcgc	27	22292087
		gaggagauguggaggggugccaagcg	26	22292087
		aucgaggagauguggaggggugcca	25	22292087
		ucgaggagauguggaggggugccaa	25	22292087
		gaggagauguggaggggugccaagc	25	22292087
		aucgaggagauguggaggggugcc	24	22292087
		ucgaggagauguggaggggugcca	24	22292087
		gaggagauguggaggggugccaag	24	22292087
		aucgaggagauguggaggggugc	23	22292087

		ucgaggagauguggaggggugcc	23	22292087
		gaggagauguggaggggugccaa	23	22292087
		aucgaggagauguggaggggug	22	22292087
		ucgaggagauguggaggggugc	22	22292087
		cgaggagauguggaggggugcc	22	22292087
		gaggagauguggaggggugcca	22	22292087
		aucgaggagauguggaggggu	21	22292087
		ucgaggagauguggaggggug	21	22292087
		cgaggagauguggaggggugc	21	22292087
		gaggagauguggaggggugcc	21	22292087
		ucgaggagauguggaggggu	20	22292087
		cgaggagauguggaggggug	20	22292087
		gaggagauguggaggggugc	20	22292087
		aggagauguggaggggugcc	20	22292087
		aucgaggagauguggaggg	19	22292087
		ucgaggagauguggagggg	19	22292087
		cgaggagauguggaggggu	19	22292087
		gaggagauguggaggggug	19	22292087
		aucgaggagauguggagg	18	22292087
		ucgaggagauguggaggg	18	22292087
		cgaggagauguggagggg	18	22292087
		gaggagauguggaggggu	18	22292087
		uacgcggcgcgcuucguccacg	22	26998839
21.	prv-miR-LLT2-5p	cucaucccgucagaccugcg	20	26998839,<br>25320324
		cucaucccgucagaccugcgcc	22	26998839
22.	prv-miR-LLT5-3p	ugaguggauggauggaggcgag	22	26998839,

				25320324
		augaguggauggauggaggcga	22	26998839
23.	prv-miR-LLT6-5p	cguaccgacccgccuaccagg	21	26998839, 25320324
		cguaccgacccgccuaccaggca	23	26998839
24.	prv-miR-LLT7-5p	ccgggggguugauggggauggg	22	26998839
		ccgggggguugauggggau	19	26998839, 25320324
25.	prv-miR-LLT8-5p	gugggggcgaagauuggguu	20	26998839, 25320324
		gugggggcgaagauuggguuggg	23	26998839
26.	prv-miR-LLT9-5p	aucgaggagauguggagggg	20	26998839, 25320324
		ucgaggagauguggaggggugc	22	26998839

12. Rat cytomegalovirus (RCMV)

S. No.	miRNA	miRNA Sequence	Length	PMID
1.	rcmv-miR-orilyt-1	gacggggucucgggcuccuga	21	20980502
		cccggagcucgaaacccgguucgb	24	20980502
		gacggggucucgggcuccugac	22	20980502
2.	rcmv-miR-orilyt-2	uggcucgcgucgccauggagac	22	20980502
		uggcucgcgucgccauggagaca	23	20980502
		gcucgcgucgccauggagaca	21	20980502
3.	rcmv-miR-r1-1	guaagauggaaucaccggagg	21	20980502
		guaagauggaaucaccggaggca	23	20980502
		guaagauggaaucaccggaggcu	23	20980502

		guaagauggaaucaccggaggc	22	20980502
		guaagauggaaucaccggag	20	20980502
4.	rcmv-miR-r1-2	uuucucucgugcuccgugucgc	22	20980502
		uuucucucgugcuccgugucg	21	20980502
5.	rcmv-miR-r1-3	ugaugcgggguaggggagugag	22	20980502
		ugaugcgggguaggggagugaga	23	20980502
6.	rcmv-miR-r111.1-1	cgcaccggcgucgagcacguac	22	20980502
		cgcaccggcgucgagcacguacu	23	20980502
		uaugugcucgucaccggagggub	23	20980502
		cgcaccggcgucgagcacgu	20	20980502
7.	rcmv-miR-r111.1-2	ucgaaacaacguggaacggcguu	23	20980502
		ucgaaacaacguggaacggcgu	22	20980502
		ucgaaacaacguggaacggcg	21	20980502
8.	rcmv-miR-r111.1-3	ucgggggcggucggaagguccc	21	20980502
		ucgggggcggucggaaggu	19	20980502
9.	rcmv-miR-r111.2-1	uacgugcucgacgccggugcgga	23	20980502
		uacgugcucgacgccggugcgg	22	20980502
10.	rcmv-miR-r111.2-2	cuucgagugcguguccgauagc	22	20980502
		cuucgagugcguguccgauagu	22	20980502
		cuucgagugcguguccgauag	21	20980502
11.	rcmv-miR-r111.2-3	aaucggacacccgcucgcgaagg	23	20980502
		aucggacacccgcucgcgaagga	23	20980502
		aaucggacacccgcucgcgaag	22	20980502
		ggacacccgcucgcgaagga	20	20980502
12.	rcmv-miR-r111.2-4	cccgaaacuccgucgaacgcgc	22	20980502
		cccgaaacuccgucgaacgcg	21	20980502
13.	rcmv-miR-r111.2-5	uuccacguuguuucgaggccu	21	20980502

		cacguuguuucgaggccu	18	20980502
14.	rcmv-miR-r111.2-6	uucgcggacgaucgaggaggcc	22	20980502
		uucgcggacgaucgaggaggc	21	20980502
		uucgcggacgaucgaggag	19	20980502
15.	rcmv-miR-r43.1-1	uuaucagccggcaagcacccagg	23	20980502
		uuaucagccggcaagcacccag	22	20980502
		uuaucagccggcaagcaccca	21	20980502
16.	rcmv-miR-r6-1	ucgaccucaagccguucgggac	22	20980502
		ucgaccucaagccguucgggaca	23	20980502
		ucccguccacuccgaggucggu	22	20980502
		ucgaccucaagccguucggga	21	20980502
17.	rcmv-miR-r87-1	ucgaagaacgggugcaacuc	20	20980502
		ucgaagaacgggugcaacucu	21	20980502
		gaagaacgggugcaacuc	18	20980502
18.	rcmv-miR-r91-1	ggacucggagucgucggacgcu	22	20980502
		ggacucggagucgucggacgcuu	23	20980502
		cguucgacgauccccgguccuu	23	20980502
		gacucggagucgucggacgcu	21	20980502

13. Rhesus lymphocryptovirus (RLCV)

S. No.	miRNA	miRNA Sequence	Length	PMID
1.	rlcv-miR-rl1-1-3p	**cuccgggccugaagagguugac**	22	16557291, 20219930
		cuccgggccugaagagguuga	21	20219930
		cuccgggccugaagagguug	20	20219930
2.	rlcv-miR-rl1-10-5p	**aucggguuuccgcuguacuugca**	23	16557291,

				20219930
		aucggguuuccgcuguacuugc	22	20219930
		aucggguuuccgcuguacuug	21	20219930
3.	rlcv-miR-rl1-13-5p	**ccugggcaugggcuaugaaaca**	22	16557291
		ccugggcaugggcuaugaaa	20	16557291,<br>20219930
		ccugggcaugggcuaugaa	19	20219930
4.	rlcv-miR-rl1-15-5p	**acccacccuucucgacggggca**	22	16557291,<br>20219930
		acccacccuucucgacggg	19	20219930
5.	rlcv-miR-rl1-16	**aagcaggcaugucuucauuc**	20	24257599
		caugaaacacauggccuguuccu	23	24257599
6.	rlcv-miR-rl1-17-5p	**ugauggacagcggggaagugcacu**	24	19889779,<br>20219930
		ugauggacagcggggaagugca	22	20219930
7.	rlcv-miR-rl1-18-5p	**uagaugauugggggcggcguauc**	23	20219930
		uagaugauugggggcggcguaucu	24	20219930
		uagaugauugggggcggcguau	22	19889779,<br>20219930
8.	rlcv-miR-rl1-19-3p	**aucaccacccccugucugcu**	20	20219930
		gaucaccacccccugucugcuu	22	20219930
		aucaccacccccugucugcuu	21	20219930
		gaucaccacccccugucugcu	21	19889779,<br>20219930
9.	rlcv-miR-rl1-2-3p	**uaucuuuugcgggggaauuuc**	21	20219930
		uaucuuuugcgggggaauuucca	23	16557291,<br>20219930

		uaucuuuugcgggggaauuucc	22	20219930
		uaucuuuugcgggggaauuu	20	20219930
10.	rlcv-miR-rl1-2-5p	**aaauucugccacagaagauagc**	22	16557291, 20219930
		aaauucugccacagaagauag	21	20219930
11.	rlcv-miR-rl1-21-5p	**uugauuguggggggagggcucu**	22	19889779, 20219930
		uugauuguggggggagggcu	20	20219930
12.	rlcv-miR-rl1-22-5p	**ucgugggggaaacgguggau**	21	20219930
		cgugggggaaacgguggau	20	20219930
		ucgugggggaaacggugga	20	20219930
		cgugggggaaacggugga	19	19889779, 20219930
13.	rlcv-miR-rl1-25-5p	**ugcuagacccuaaauuggaaac**	22	19889779, 20219930
		ugcuagacccuaaauuggaaa	21	20219930
		ugcuagacccuaaauuggaa	20	20219930
14.	rlcv-miR-rl1-27-5p	**gccgccuccuugguucuguc**	20	20219930
		gccgccuccuugguucugucc	21	20219930
		gccgccuccuugguucugucca	22	19889779, 20219930
15.	rlcv-miR-rl1-28-5p	**uggaaggcguugcuuucagcug**	22	19889779, 20219930
		uggaaggcguugcuuucag	19	20219930
16.	rlcv-miR-rl1-29-5p	**ccagucccagcagacaaaac**	20	20219930
		ccagucccagcagacaaaaca	21	19889779, 20219930

17.	rlcv-miR-rl1-3	**ucgcaccucgccgucucuacugc**	23	24257599
		ucgcaccucgccgucucuacugcu	24	16557291, 20219930
		cgguggagaccgcgggugcggug	23	24257599
18.	rlcv-miR-rl1-30-5p	**accgguccuuugagguacgaaac**	23	20219930
		accgguccuuugagguacgaaaca	24	19889779, 20219930
		accgguccuuugagguacgaaa	22	20219930
19.	rlcv-miR-rl1-33-3p	**aaagagcaaaugggggaauagc**	23	20219930
		aaagagcaaaugggggaauag	22	19889779, 20219930
20.	rlcv-miR-rl1-34-3p	**uuagcccgaaccgcuauccaua**	22	20219930
		guuagcccgaaccgcuauccaua	23	20219930
		uuagcccgaaccgcuauccau	21	20219930
21.	rlcv-miR-rl1-34-5p	**uagauggcguaacaggcaacu**	21	20219930
		uagauggcguaacaggcaacuu	22	20219930
22.	rlcv-miR-rl1-35-3p	**uguaacuugucgugggacggg**	21	20219930
		uguaacuugucgugggacgggca	23	20219930
		uguaacuugucgugggacgggc	22	20219930
		guaacuugucgugggacgggca	22	20219930
		uguaacuugucgugggacgg	20	20219930
		guaacuugucgugggacggg	20	20219930
		uguaacuugucgugggacg	19	20219930
		uguaacuugucgugggacgggcau	24	20219930
23.	rlcv-miR-rl1-35-5p	**caccggcucacagcuaguacgg**	22	20219930
		caccggcucacagcuaguacg	21	20219930
24.	rlcv-miR-rl1-7-3p	**agugggccuguuucccucaca**	21	16557291,

				20219930
		agugggccuguuucccucac	20	20219930
25.	rlcv-miR-rl1-8-3p	**ggugggccgcaguucaccuaca**	22	16557291, 20219930
		ggugggccgcaguucaccuac	21	20219930

14. Rhesus rhadinovirus (RRV)

S. No.	miRNA	miRNA Sequence	Length	PMID
1.	rrv-miR-rr1-1-3p	**gccaccgaggaugcggucaau**	21	20655562
		ggccaccgaggaugcggu	18	20655562, 17451774
2.	rrv-miR-rr1-1-5p	cgaucgcaccuuuggccggc	20	20655562, 17451774
		gaucgcaccuuuggccggc	19	20655562
3.	rrv-miR-rr1-11-3p	**agugggucucggguuuggggga**	21	20655562
		agugggucucggguuuggggaa	22	20655562
		agugggucucggguuuggggaau	23	20655562
4.	rrv-miR-rr1-12-3p	**auuucguuuuuagauucccuaa**	22	20655562
		auuucguuuuuagauucccuaau	23	20655562
		auuucguuuuuagauucccua	21	20655562
		auuucguuuuuagauuccccu	20	20655562
5.	rrv-miR-rr1-12-5p	**uagggaacuaaagacaauuu**	20	20655562
		uagggaacuaaagacaauu	19	20655562
		uagggaacuaaagacaauuuc	21	20655562
6.	rrv-miR-rr1-13-3p	**uaguaaccugacagcaguuag**	21	20655562
		uaguaaccugacagcaguuagu	22	20655562
		uaguaaccugacagcaguua	20	20655562

		uaguaaccugacagcaguu	19	20655562
7.	rrv-miR-rr1-13-5p	**uaauugcaguugguaugcuacu**	22	20655562
		uaauugcaguugguaugcuac	21	20655562
		aauugcaguugguaugcuac	20	20655562
		auugcaguugguaugcuac	19	20655562
		uaauugcaguugguaugc	18	20655562
8.	rrv-miR-rr1-14	**aagggacccaaagacacagu**	20	20655562
		aagggacccaaagacacaguc	21	20655562
		aagggacccaaagacacag	19	20655562
		aagggacccaaagacaca	18	20655562
9.	rrv-miR-rr1-15-3p	**caguguuguucgugaacguaga**	22	20655562
		caguguuguucgugaacguag	21	20655562
10.	rrv-miR-rr1-15-5p	**ugcggucaccaacaacacuauu**	22	20655562
		ugcggucaccaacaacacuau	21	20655562
		ugcggucaccaacaacacua	20	20655562
		ugcggucaccaacaacacu	19	20655562
		gcggucaccaacaacacu	18	20655562
11.	rrv-miR-rr1-2	**auacggcgcugcacgguugga**	21	17451774
		uauacggcgcugcacgguugga	22	20655562
		uauacggcgcugcacgguugg	21	20655562
		uauacggcgcugcacgguu	19	20655562
12.	rrv-miR-rr1-3-3p	caggguuugcgacagacac	19	20655562
		aggguuugcgacagacac	18	20655562
13.	rrv-miR-rr1-3/ rrv-miR-rr1-3-5p	**cccgaugagcaguuagucc**	19	17451774
		guguuuagucgcgcucccuguu	22	20655562
		guguuuagucgcgcucccugu	21	20655562
14.	rrv-miR-rr1-4*/ rrv-miR-rr1-	**cucguuaaccgcccucccgaga**	22	17451774

	4-3p	cucguuaaccgcccuccugaga	22	20655562
		cucguuaaccgcccuccugag	21	20655562
		cucguuaaccgcccuccuga	20	20655562
15.	rrv-miR-rr1-4-5p	**uggggagggcggucagcgcgcg**	22	20655562
		cucguuaaccgcccucccgaga	22	20655562
		cucguuaaccgcccucccga	20	20655562
		guuaaccgcccucccgaga	19	20655562
		uggggagggcggucagcgc	19	20655562
16.	rrv-miR-rr1-5-3p	ggcguguucuuuggauucc	19	20655562
		ggcguguucuuuggauuc	18	20655562
17.	rrv-miR-rr1-5/ rrv-miR-rr1-5-5p	**ccggaacccaaagacacgugcccg**	24	17451774
		cccggaacccaaagacacgugcc	23	20655562
		cccggaacccaaagacacgugc	22	20655562
		cccggaacccaaagacac	18	20655562
18.	rrv-miR-rr1-6-3p	**cgauguacgcccuuucgcagu**	21	20655562
		gauguacgcccuuucgcagu	20	20655562
		cgauguacgcccuuucgcag	20	20655562
19.	rrv-miR-rr1-6/ rrv-miR-rr1-6-5p	**cgcggaaaggugugcacaucgua**	23	17451774
		cgcggaaaggugugcacaucg	21	20655562
		cgcggaaaggugugcaca	18	20655562
20.	rrv-miR-rr1-7-3p	**cgcacgucgauugcucucuag**	21	17451774
		cgcacgucgauugcucucuagg	22	20655562
		cgcacgucgauugcucucu	19	20655562
		cgcacgucgauugcucuc	18	20655562
21.	rrv-miR-rr1-7-5p	**uggagagcaguuaacgugcguuc**	23	17451774
		uggagagcaguuaacgugcguu	22	20655562
		ggagagcaguuaacgugcguu	21	20655562

		gagagcaguuaacgugcguu	20	20655562
		uggagagcaguuaacgugc	19	20655562
		uggagagcaguuaacgug	18	20655562
22.	rrv-miR-rr1-8-3p	aaagugcucacaagacauccc	21	20655562
		aaagugcucacaagacaucc	20	20655562
		aaagugcucacaagacauc	19	20655562
23.	rrv-miR-rr1-8-5p	**gaugucuugcgggcacucucc**	21	20655562
		gaugucuugcgggcacucuc	20	20655562
		gaugucuugcgggcacuc	18	20655562
24.	rrv-miR-rr1-9-3p	**gcggucaucagaacauaucacc**	22	20655562
		gcggucaucagaacauaucac	21	20655562
		gcggucaucagaacauauc	19	20655562
		cggucaucagaacauauca	19	20655562
		gcggucaucagaacauau	18	20655562
		ggcggucaucagaacaua	18	20655562
25.	rrv-miR-rr1-9-5p	**aauauguuccgcugacaccgcug**	23	20655562
		gaauauguuccgcugacaccgc	22	20655562
		aauauguuccgcugacaccgc	21	20655562
		aauauguuccgcugacacc	19	20655562
		aauauguuccgcugacac	18	20655562
